# Full Control of Plasmonic Nanocavities Using Gold Decahedra‐on‐Mirror Constructs with Monodisperse Facets

**DOI:** 10.1002/advs.202207178

**Published:** 2023-02-03

**Authors:** Shu Hu, Eoin Elliott, Ana Sánchez‐Iglesias, Junyang Huang, Chenyang Guo, Yidong Hou, Marlous Kamp, Eric S. A. Goerlitzer, Kalun Bedingfield, Bart de Nijs, Jialong Peng, Angela Demetriadou, Luis M. Liz‐Marzán, Jeremy J. Baumberg

**Affiliations:** ^1^ Nanophotonics Centre Department of Physics Cavendish Laboratory University of Cambridge Cambridge England CB3 0HE UK; ^2^ CIC biomaGUNE Basque Research and Technology Alliance (BRTA) Paseo de Miramón 194 Donostia‐San Sebastián 20014 Spain; ^3^ School of Physics and Astronomy University of Birmingham Birmingham B15 2TT UK; ^4^ Ikerbasque Basque Foundation for Science Bilbao 43009 Spain; ^5^ Present address: College of Advanced Interdisciplinary Studies and Hunan Provincial Key Laboratory of Novel Nano‐Optoelectronic Information Materials and Devices National University of Defense Technology Changsha 410073 P. R. China

**Keywords:** gold decahedron, metal nanoparticle, nanophotonics, picocavity, plasmonic nanocavity, surface‐enhanced Raman spectroscopy

## Abstract

Bottom‐up assembly of nanoparticle‐on‐mirror (NPoM) nanocavities enables precise inter‐metal gap control down to ≈ 0.4 nm for confining light to sub‐nanometer scales, thereby opening opportunities for developing innovative nanophotonic devices. However limited understanding, prediction, and optimization of light coupling and the difficulty of controlling nanoparticle facet shapes restricts the use of such building blocks. Here, an ultraprecise symmetry‐breaking plasmonic nanocavity based on gold nanodecahedra is presented, to form the nanodecahedron‐on‐mirror (NDoM) which shows highly consistent cavity modes and fields. By characterizing > 20 000 individual NDoMs, the variability of light in/output coupling is thoroughly explored and a set of robust higher‐order plasmonic whispering gallery modes uniquely localized at the edges of the triangular facet in contact with the metallic substrate is found. Assisted by quasinormal mode simulations, systematic elaboration of NDoMs is proposed to give nanocavities with near hundred‐fold enhanced radiative efficiencies. Such systematically designed and precisely‐assembled metallic nanocavities will find broad application in nanophotonic devices, optomechanics, and surface science.

## Graphical abstract

1 nm high metallic optical cavities of precise size and shape are self‐assembled, which exhibit highly‐consistent modes and greatly improve directional light emission, thereby enabling widespread application in optoelectronics through extreme light confinement.

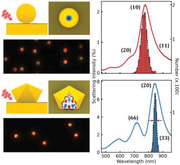

## 1 Introduction

The unique chemical and physical properties of noble metal nanostructures have given rise to key developments in fundamental science and technology, such as nanocatalysis,^[^ ^1^, ^2^, ^3^ ^]^ nanooptics,^[^ ^4^, ^5^ ^]^ and biosensors.^[^ ^6^, ^7^ ^]^ For enhanced light‐matter interactions, metal nanostructures of Au, Ag, and Cu supporting collective free electron oscillations are the most attractive materials for fabricating metallic nanocavities.^[^ ^5^, ^8^ ^]^ Extreme light confinement to the subwavelength or even atomic scale has been achieved using such nanocavities, generating huge electromagnetic (EM) field enhancements inside metallic nanogaps that can access atomic‐scale resolution and sensitivities down to individual bond vibrations.^[^ ^9^, ^10^, ^11^, ^12^ ^]^ Such achievements have driven a persistent effort to fabricate robust nanocavities with precise spectral modes and high radiation efficiencies, which facilitate the development of diverse applications such as surface‐enhanced Raman scattering (SERS),^[^ ^13^, ^14^ ^]^ quantum computing,^[^ ^15^ ^]^ room temperature polaritonic nanolasers,^[^ ^16^ ^]^ and single photon sources.^[^ ^17^ ^]^ However, such an extreme EM field confinement requires creating consistent inter‐metal gaps of less than 5 nm width, which is challenging for conventional top‐down lithographic techniques, for instance in dipole antennas,^[^ ^18^ ^]^ patch antennas,^[^ ^19^ ^]^ and metal‐insulator‐metal nanocavities (MIMs).^[^ ^20^ ^]^

An alternative and very successful approach has been to define the gap spacing using atomic layers (such as WSe_2_) or self‐assembled molecular monolayers (SAMs) onto which a nanoparticle (NP) is then assembled to form the MIM nanogap. Using typical 40–150 nm diameter Au or Ag NPs (which are facetted according to the Wulff construction), nanoparticle‐on‐mirror (NPoM) constructs are readily obtained using bottom‐up large‐scale methods, with controllable gap sizes down to sub‐nanometer scales.^[^ ^5^, ^21^, ^22^, ^23^ ^]^ Massive EM field enhancements *E* > 600 have been achieved, sufficient to detect single molecules.^[^ ^10^, ^24^ ^]^ However, in such symmetric antennas the dominant vertically‐polarized gap modes radiate at high angles (≈ 55°) making them difficult to fully collect in air using objective lenses with numerical aperture NA<1.^[^ ^5^ ^]^

More critically, signal enhancements vary significantly between NPoMs, even with the same diameter, because the NPoM optical properties depend on even atom‐scale variations of the facets, edges, and vertices when the metallic gaps are so thin.^[^ ^25^, ^26^ ^]^ This complicates the quantitative link between light input/output coupling and nanocavity structure, as well as the subtle correlations between elastic (dark‐field) and inelastic (Raman) spectra, which requires a full understanding (and then full control). A recent theoretical study using quasinormal modes (QNMs) showed that the exact shape and size of the nanogap facet is a crucial factor in the variability of nanocavities.^[^ ^25^ ^]^ Practically however, it is hard to characterize each structural parameter that is involved. The “spherical” citrate‐capped nanoparticles commonly synthesized have a broad size distribution (typically 10%) and expose complicated polyhedral geometries instead of ideal spheres. Progress in shape‐controlled synthesis now creates noble metal nanocrystals of better‐defined morphology and uniform size.^[^ ^27^ ^]^ However unlike the easily‐removed citrate ligands, these require strong binding ligands such as hexadecyltrimethyl‐ammonium bromide (CTAB), hexadecyltrimethylammonium chloride (CTAC), or polyvinylpyrrolidone (PVP) that form surface bilayers >2 nm thick, significantly enlarging the metallic nanogap and restricting accessible gap chemistries.

**Figure 1:**
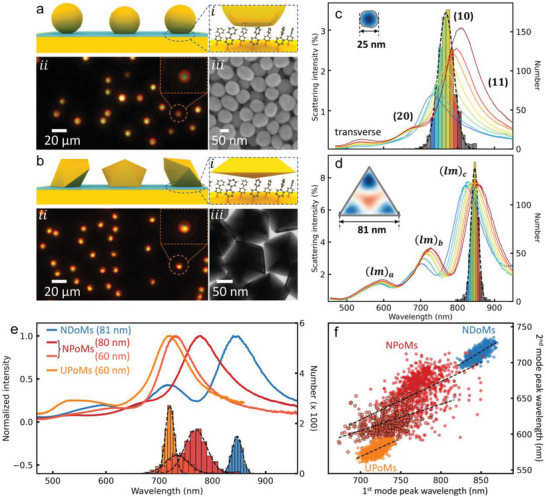
Dark‐field (DF) characterization of nanoparticle‐on‐mirror (NPoM) and nanodecahedron‐on‐mirror (NDoM) nanocavities. a,b) Schematics, DF and electron microscopy images of a) NPoMs and b) NDoMs. c,d) Histograms of the dominant cavity mode wavelengths, together with average spectra from each bin of c) *D* = 80 nm NPoMs with facet (typically 25nm), and d) *R*_edge_= 81 nm NDoMs. Insets show simulated near‐fields of (10) and (*lm*)_c_ modes. e) Wavelength distributions of the dominant mode for 60 nm NPoMs (light red), 80 nm NPoMs (dark red), 81 nm NDoMs (blue), and 60 nm ultraspherical NPoMs (UPoMs, orange). Corresponding average spectra for all the nanocavities in the center (most common) bin. f) Wavelength correlation of two main cavity modes, (20) versus (10) for NPoMs (60, 80 nm)/UPoMs (60 nm), (*lm*)_b_ versus (*lm*)_c_ modes for NDoMs of 81 nm, color labeled as in (e).

In this work, we present a big data approach to analyze the plasmonic scattering and SERS spectra of more than 20 000 individual nanocavities, using a sophisticated automated particle tracking rig. The results systematically reveal the spectral and field enhancement variations induced by nanocavity morphology and how these can affect light in/out coupling. To suppress such variations and enhance light coupling, we introduce a symmetry‐breaking antenna built from a nanodecahedron on mirror (NDoM) configuration. These are constructed using Au nanodecahedra which are carefully synthesized and then citrate‐stabilized using ligand exchange.^[^ ^28^ ^]^ These nanodecahedra are of extremely high consistency in size and morphology, each comprising 10 well‐defined triangular Au (111) monodisperse facets (**Figure** **1**). We thus now control four key parameters: crystal plane, facet shape, facet size, and NP height. Indeed NDoMs are found to exhibit a peculiar asymmetric radiation pattern but show greatly improved consistency with two‐fold narrower spectral distribution and five‐fold better light coupling distribution. However, unaccounted variation still remains when correlating the cavity modes with SERS, which is thoroughly explored. NDoMs support higher‐order cavity modes that generate strong field enhancements and can be easily tuned across the whole visible region even with extremely thin gaps *d* ≤  1.5 nm. QNM analysis with COMSOL simulations gives a comprehensive description of the NDoM nanocavity modes, which are shown to match well with the experimental spectra. Finally, general guidelines are proposed and carried out for designing and fabricating robust high‐efficiency nanocavities. An elaborated NDoM nanocavity is devised, which enables SERS signal enhancements up to two orders of magnitude higher than those from NPoMs (reaching > 10^6^ counts mW^−1^ s^−1^), for the same size of facet and metallic gap.

## 2 Results and Discussion

### 2.1 Comparing Plasmonic Nanocavity Modes of NPoMs and NDoMs

In our protocol, NPoMs are fabricated by assembling *D*  =  80 nm diameter citrate‐capped Au NPs on top of template‐stripped Au films precoated with biphenyl‐4‐thiol (BPT) molecular SAMs (*d* ≈ 1.5 nm spacer, Figure 1a(i)). The dark field (DF) optical microscopy images of these NPoMs (Figure 1a(ii)) exhibit color patterns indicating the resonant mode wavelengths and their far‐field radiation. Each NPoM gives a circularly‐symmetric pattern with a green spot surrounded by a red doughnut‐shaped ring. The green spot arises from the uncoupled transverse mode, whereas the ring is the dominant coupled mode that radiates to high angles symmetrically.^[^ ^21^ ^]^ Differences in the DF patterns of different NPoMs (intensity, size, and color in Figure 1a(ii)) indicate morphology and size variations among citrate NPs, as confirmed by SEM characterization (Figure 1a(iii)). To characterize the mode distribution, plasmonic scattering spectra of more than 1000 individual NPoMs are measured and sorted (Figure 1c). Due to objective lens chromatic aberration across *λ* =  450–1000 nm, each NPoM scattering spectrum must be carefully reconstructed from a series collected while scanning through the focus (Figure S1a–c, Supporting Information and Experimental Section).^[^ ^29^ ^]^

The NPoMs support several modes, including the transverse mode (≈ 530 nm) and coupled modes (labeled according to ref. [25]): with (10) at ≈ 770 nm, (11) at ≈ 850 nm, and (20) at ≈ 650 nm. Histograms are sorted by the dominant mode wavelength *λ*_10_ and scattering spectra averaged within each histogram bin of *λ*_10_ (corresponding colors in Figure 1c). The distribution of *λ*_10_ is spread across >100 nm with large correlated changes in scattering intensity. The gap size is set by a highly consistent BPT SAM (see below) with a difference of only 1 Å in height as shown by scanning tunneling microscopy (STM).^[^ ^30^ ^]^ The distribution is thus set by remaining variations in diameter, facet size (contact area), and exact NP morphology.^[^ ^25^ ^]^ Indeed, transverse and (20) modes that are sensitive to NP diameter and facet size closely follow the change in average ${\bar{\lambda }}_{10}$,^[^ ^25^, ^26^ ^]^ with intensities increasing as their wavelengths redshift. A linear correlation is found when plotting ${\bar{\lambda }}_{20}$ versus ${\bar{\lambda }}_{10}$ (Figure 1f; and Figure S2, Supporting Information), which is expected from NP size gradation. However, a large variation is seen in *λ*_20_ for NPoMs with the same *λ*_10_ (Figure 1f; and Figure S3a, Supporting Information). The intensity distributions of the different modes also show weak correlations, even when selecting only those at the same *λ*_10_ and (10) scattering intensity (Figure S3b, Supporting Information). This indicates that facets vary considerably even for NPoMs of similar size. Similar results are found when using NP batches of different average diameter (Figure 1e,f). Therefore, NPoMs have quasicircular facets but which vary in both shape and diameter.^[^ ^22^ ^]^

To better constrain the nanocavity modes in metallic nanocavities, well‐defined nanoparticles are essential. Here, a modified seeded‐growth method enables extremely high synthetic yields of precisely‐defined nanocrystals as Au nanodecahedra (NDs).^[^ ^28^ ^]^ Their identical size and shape can be clearly observed in transmission electron microscopy (TEM) images (Figure 1b(iii)). These Au NDs are then used to make a new type of NDoM metallic nanocavity with BPT SAMs as the spacer (Figure 1b). Their canted orientation on the mirror breaks the symmetry of far‐field radiation so they give horseshoe‐shaped patterns in DF images (Figure 1b(ii)), very different from NPoMs. The orientation of each DF horseshoe gives the precise orientation of each nanodecahedron on the surface (Figure S4, Supporting Information), while their DF patterns are near‐identical because of their well‐defined facets.

To reveal the modified plasmonic modes of NDoMs, scattering spectra from more than a thousand individual NDoMs are acquired, reconstructed, and sorted (Figure 1d; and Figure S1d–f, Supporting Information). Three new cavity modes can be observed radiating at high angles (Figure S5, Supporting Information), labeled as (*lm*)_a,b, c_ since they originate from circular symmetry modes indexed by radial and azimuthal integers *l*, *m*. The near field map (inset) of the strongest mode (*lm*)_c_ shows the EM field is more tightly confined around the edges and vertices of the large facet rather than at its center, as is the case for NPoMs (Figure 1c inset). The average scattering intensity of NDoMs is found to be more than three times higher than NPoMs with the same Au volume (*D* ≃ *R*_edge_, Figure S6, Supporting Information), showing the higher radiative coupling efficiency of NDoM cavity modes. More significantly, the wavelength distribution full‐width at half‐maximum (FWHM) of mode (*lm*)_c_ is now half that of NPoMs, even though the (*lm*)_c_ mode is confined to the facet edges and should be more sensitive than the NPoM (10) mode (see below). This highlights the extremely consistent facet dimensions and shape of NDoMs. The Q‐factor of the resonant plasmon modes is also increased in NDoMs compared to NPoMs.

Comparing DF spectra with the same main peak wavelength shows dramatically more consistency for NDoMs than NPoMs (Figure S3, Supporting Information), particularly in the strength and spectral position of adjacent peaks. To further confirm that facet variability is important, ultraspherical Au NPs are synthesized with CTAC (see the Experimental Section) and subsequent ligand exchange to citrate. Such ultraspherical particles are metastable (to Wulff reconstruction) and form initially small facets when binding to the mirror that build ultraspherical nanoparticle‐on‐mirror (UPoM) constructs, but which can vary from batch to batch (Figure S7, Supporting Information). Compared to NPoMs of the same size, the DF images are more consistent (Figure S8, Supporting Information) and the *λ*_10_ distribution is as narrow as the one for NDoMs (Figure 1e). The smaller contact facet of UPoMs is seen in their blueshifted *λ*_20_ mode compared to similar sized NPoMs (Figure 1f). While UPoMs and NDoMs both better control the nanocavity modes, only the faceted NDoMs are stable – the large van der Waals attraction between NP and mirror, *F*_VdW_ = *AD*/6*d* ^2^ ≈ 3 nN for Au Hamaker constant *A* ≈ 1 eV, corresponds to 100 atmosphere pressures over a 20 nm wide facet, which reconstructs each ultraspherical NP.

The above results show that optical consistency of metallic nanocavities can be achieved by carefully controlling the facet and shape of the NPs, delivering a highly effective strategy to precisely control the nanocavity mode spectrum and fields. However we note that a remaining uncontrolled component of order ± 1% remains in the mode spectrum (Δ *λ*_c_ = ± 10 nm from Figure 1f). This may be due to small shape differences, such as rounding of the fivefold vertex at the bottom facet triangle corner.

### 2.2 Light in/out Coupling from NDoMs and NPoMs

**Figure 2:**
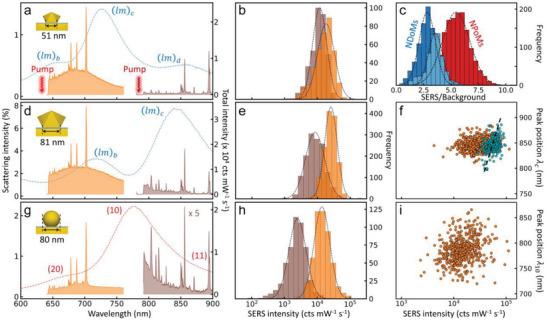
Characterization of light in/out coupling of NDoMs and NPoMs. a,d,g) Average DF scattering and two‐wavelength SERS spectra of BPT, excited with lasers at 633 nm (orange) and 785 nm (brown), using a) 51 nm NDoMs, d) 81 nm NDoMs, and g) 80 nm NPoMs (see insets). b,e,h) Histograms of 1550 cm^−1^ SERS intensities from b) 51 nm NDoMs, e) 81 nm NDoMs, and h) 80 nm NPoMs (colors as in (a)). c) Distribution of intensity ratio of molecular SERS to background Au light emission extracted from 81 nm NDoMs (blue), 51 nm NDoMs (light blue), and 80 nm NPoMs (red); pump laser at 633 nm. f,i) Correlation of (*lm*)_c_ or (10) mode wavelength with 1550 cm^−1^ SERS intensity (background subtracted) for f) 81 nm NDoMs and i) 80 nm NPoMs. Cyan points show correlation for 633 nm excitation, after compensating measured polarization and chiral effects on each NPoM (see text). Axes show same ranges.

To quantify the coupling of light from free space into and out of metallic nanocavities, two‐wavelength SERS is used to characterize the field enhancements and their correlation with the cavity modes.^[^ ^31^ ^]^ In this technique, simultaneous Raman pumping at 633 and 785 nm (which have different near‐field distributions and coupling) are compared for each construct. SERS spectra and corresponding DF plasmonic spectra for more than 3000 individual NDoMs and NPoMs are acquired and sorted by the strongest mode (**Figure** **2**). The average DF scattering (dashed) and SERS (orange, brown) spectra of the histogram central bin are compared (Figure 2a,d,g), showing that the 633 and 785 nm lasers excite/out‐couple with (*lm*)_b,c_ modes, respectively, for 81 nm NDoMs (Figure 2d).

The SERS intensity of the BPT aromatic ring stretch (1550 cm^−1^) in each NDoM is extracted (normalized using silicon signals, see the Experimental Section) to give an intensity histogram (Figure 2e). This shows that 785 nm excitation gives weaker SERS with 1.5‐fold broader variability than with the 633 nm laser (detailed comparison in Figure S9, Supporting Information), indicating that (*lm*)_b_ and (*lm*)_c_ modes have similar overall efficiency. To further explore the NDoM cavity modes, smaller nanodecahedra of 51 nm are similarly characterized (Figure 2a). The spectral positions of (*lm*)_b,c_ blueshift, as expected, while a new mode labeled (*lm*)_d_ emerges at 850 nm (for 81 nm NDoMs it is > 950 nm, beyond our current detection range). Despite the fivefold weaker scattering of the (*lm*)_d_ mode, similar SERS intensities are observed when the same vibration out‐couples with either (*lm*)_c_ (Figure 2b orange) and (*lm*)_d_ (Figure 2b brown), which suggests stronger out‐coupling of (*lm*)_d_. The same characterization is applied to a set of 80 nm NPoMs (Figure 2g,h). Stronger SERS signals are observed when exciting/out‐coupling through the (20) (Figure 2g,h orange) mode compared to ≈ 6 times weaker SERS from the (10) mode (Figure 2g,h brown). Overall, NDoMs have slightly higher SERS intensity when excited at both 633 and 785 nm, compared to NPoMs. This indicates NDoMs provide better consistency but do not significantly improve the light in‐/out‐coupling.

To evaluate the effect of each cavity mode, the intensity ratios of molecular to background scattering (under the SERS peaks, either from Au electronic Raman scattering, ERS,^[^ ^32^ ^]^ or Au photoluminescence^[^ ^33^ ^]^), are extracted from 633 nm SERS spectra of NPoMs (red) and NDoMs (blue‐81 nm, light blue‐51 nm, Figure 2c). As expected, this ratio is more consistent for NDoMs. Interestingly, the Au light emission does not exactly track the scattering spectra of the nanocavity. This discrepancy originates from variable redshifts of near‐ to far‐field resonances,^[^ ^34^ ^]^ which are cavity mode dependent and affected by the exact geometry of the nanocavity. Examining the correlation of SERS versus *λ*_10_ shows large and random dispersion for NPoMs (Figure 2i). By contrast, the SERS intensity is well constrained with *λ*_c_ in NDoMs (Figure 2f) due to the accurate control over ND morphology. Nevertheless, a fivefold variability in SERS is still evident, which is not ideal. In addition, the SERS intensity is barely correlated with *λ*_c_ (Figure 2f) or *λ*_b_ (Figure S10a, Supporting Information), even if the excitation or out‐coupling are in resonance. Similar results are obtained for 51 nm NDoMs (Figure S10b, Supporting Information), where *λ*_b_ pumps directly at the resonance position.

### 2.3 SERS Signal Variability from NDoMs

**Figure 3:**
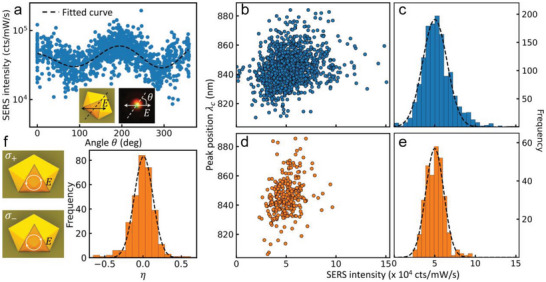
Linearly and circularly polarized SERS excitation of 81 nm NDoMs for a–c) linear and d–f) circularly polarized 633 nm laser excitation. a) Polarization‐dependent SERS intensity from NDoMs, dashed line is a sinusoidal fit. b,e) Correlation of 1550 cm^−1^ SERS intensity with (*lm*)_c_ mode wavelength and c,f) SERS intensity distribution after excluding b,c) polarization and d,e) chirality effects. f) Net chirality ratio *η* = (*I*_+_ − *I*_−_) /(*I*_+_ + *I*_−_) of right‐handed to left‐handed pumped circularly polarized SERS intensities.

One hypothesis for the origin of the residual uncertainties is additional polarization‐dependent coupling due to the symmetry‐breaking of NDoMs. Therefore, we perform polarized SERS measurements on single NDoMs, where a SERS intensity modulation of ± 35% is typically seen as the laser linear polarisation is rotated (Figure S11c, Supporting Information). The SERS intensity is maximized (minimized) when the laser *E* is polarized along the head (side) of each horseshoe pattern respectively (Figure S9a,b, Supporting Information). To exclude polarization effects in SERS from NDoMs and avoid time‐consuming polarized SERS measurements on each one, we extract the angle *θ* of each horseshoe pattern referenced to the laser polarization and correlate this with SERS intensity (**Figure** **3**a). A clear polarization angle‐dependence of SERS is seen, with intensity ratio of maximum to minimum ≈ 1.6 from the sin ^2^ *θ* fit (Figure 3a, black dashed line). This ratio matches that obtained from polarization‐rotation measurements on individual NDoMs, showing that the laser is preferentially coupled into these nanocavities when its polarization is along the symmetry line of the bottom facet (through the fivefold apex point). However, this is not the only variability, and the distribution of remaining SERS fluctuations is still ± 60% (Figure 3b,c) after normalizing out polarization effects for each NDoM (Figure S12, Supporting Information). We thus explore additional optical anisotropies that can be responsible for this effect.

To minimize uncontrolled circular dichroism optical effects that can induce signal variability,^[^ ^35^ ^]^ we acquire SERS spectra from single NDoMs exciting with left‐handed (LCP *σ*_+_) and right‐handed (RCP *σ*_−_) circularly polarized light (Figure 3f, left). In this case, equal linear polarizations along and across the horseshoe are incident, giving more uniform near‐field excitation. We still find significant variation between LCP SERS *I*_+_ and RCP *I*_−_, with *η* = (*I*_+_ − *I*_−_) /(*I*_+_ + *I*_−_) ranging from −0.5 to 0.5 (Figure 3f). This clearly indicates some NDoMs respond differently to LCP and RCP excitation, and that NDoMs have a random chiral response. Since the NDoM geometry has an axis of symmetry, this is surprising. It must arise from subtle structural imperfections of the metal nanocrystals,^[^ ^36^, ^37^ ^]^ likely through the seeded‐growth process, and which are amplified by the intense light confinement, but which are not easily observed in electron microscopy. Plotting the correlation of mode *λ*_c_ with the average LCP/RCP SERS intensity, $\bar{I}=\frac{1}{2}\;\left(I_{+}+I_{-}\right)$, shows a larger (>3‐fold) suppression of SERS variation (Figure 3e). This also now gives a much tighter correlation of SERS intensity with cavity mode (Figure 2f, cyan) compared with NPoMs (Figure 2i, orange). The residual correlation of SERS intensity with *λ*_c_ (Figure 3d) comes from small changes in ND size, which change optical in‐coupling, and can thus be normalized out. This allows systematic prediction of optical performance for these plasmonic nanocavities. Remaining uncontrolled SERS signal variations may come from subtle curvature variation of the facet edges or the excitation of additional dark modes, which require further investigation.

### 2.4 Size‐Dependent Effect of NDoMs

**Figure 4:**
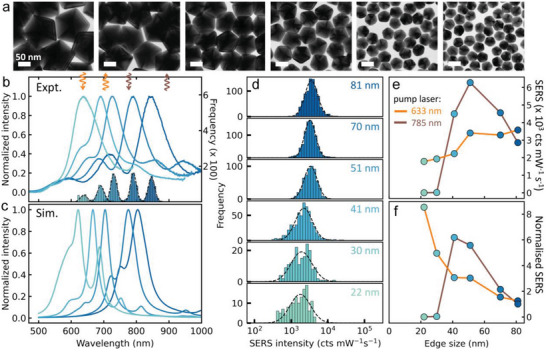
Size‐dependent tuning of plasmonic modes and SERS of NDoMs. a) TEM images of gold decahedra with edge lengths of 81, 70, 51, 41, 30, and 22 nm. b) Histograms of *λ*_c_ for each size with averaged DF spectra from center bin (colors as d), from left to right sizes of 30, 41, 51, 70, 81 nm). c) Simulated scattering spectra of NDoMs of the same size as in (b). d) SERS (1550 cm^−1^) intensity distribution of NDoMs of each size, excited at 633 nm. e,f) SERS intensity for increasing NDoM size [e) absolute, or f) normalized by facet area], excited with 633 and 785 nm lasers, as labeled.

To explore this size dependence of NDoM cavity modes, a graded set of nanodecahedra sizes are synthesized. These are highly uniform (**Figure** **4**a, more details in Figure S13, Supporting Information), with similar DF patterns observed for each size (Figure S14, Supporting Information). The scattering spectra of >1000 NDoMs of each size are acquired and sorted by the dominant mode *λ*_c_ (Figure 4b). In NDoMs, four bands *λ*_a − d_ are observed, with *λ*_d_ > 1000 nm for 81 nm NDoMs and *λ*_a − d_ overlapping for 30 nm NDoMs. The mode wavelengths tune across a broad range from visible to near‐infrared, even with extremely thin nanogaps of *d* ≈ 1.5 nm. Moreover, the energy distribution for each size is well separated (Figure 4b, histograms), in sharp contrast to NPoMs (Figure 1e). COMSOL simulations are performed to obtain the predicted NDoM scattering spectra for corresponding sizes (Figure 4c). These simulated spectra match well their experimental counterparts, showing similar spectral features and size‐dependent wavelength tuning.

To evaluate their efficiencies, two‐wavelength SERS is performed on each NDoM size. The 1550 cm^−1^ SERS intensity from each individual NDoM is extracted (Figure 4d, 633 nm; Figure S15 (Supporting Information) for 785 nm excitation). Average SERS intensities (peak of dashed curves, Figure 4d) are found to gradually increase with size (Figure 4e) for 633 nm excitation, while instead showing a clear maximum at 51 for 785 nm excitation. Normalization of the signals by facet area to compare SERS per molecule (Figure 4f) shows highest 633 nm SERS per molecule for small sizes, due to the resonance of *λ*_c_ near the laser wavelength. For 785 nm SERS, which gives a higher overall efficiency, optimal SERS is obtained when the pump is blue‐detuned from *λ*_c_, which is completely different to what happens for NPoMs (Figure 2i). In addition, the variability of 633 nm SERS clearly increases with decreasing size (Figure 4d), which is opposite for 785 nm SERS (Figure S15, Supporting Information). To explain these behaviors it is necessary to characterize the near‐field distributions under the gap facet for each mode.

### 2.5 Quasinormal Mode Description of NDoMs

**Figure 5:**
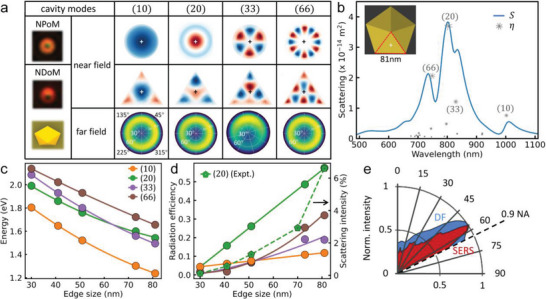
COMSOL simulations and angular emission measurement of NDoMs. a) Simulated near/far field profiles and DF images of NPoMs versus NDoMs (nanodecahedron and facet orientations shown in inset to a,b), respectively). b) Simulated scattering spectra of 81 nm NDoM with cavity modes labeled as in (a), gray points give relative radiative efficiency of each mode. Inset shows a Au decahedron with lowest facet dashed, oriented as in (a). c,d) Size‐dependent c) mode energy and d) radiative efficiency of main NDoM cavity modes. e) Measured emission angles of scattering (blue) and SERS (red) from a NDoM using *k*‐space angular spectroscopy.

To better understand the NDoM mode spectrum, a QNM approach is used, which provides a complete description (**Figure** **5**). In 81 nm NDoMs, many modes exist for *λ* < 1100 nm (Figure 5b, gray stars). However, only four modes possess significant radiation efficiency, labeled (10), (20), (33), and (66) by analogy with their field patterns under the nominally‐circular facets of NPoMs (Figure 5a). Both (20) and (33) modes overlap to give the dominant (*lm*)_c_ mode, while (66) =(*lm*)_b_ and (10) =(*lm*)_d_. Each cavity mode resonance redshifts with increasing size, while their radiative efficiency increases (Figure 5c,d), closely matching the experimental results (Figure S16, Supporting Information) and confirming their correct identification (Figure 4b). Compared to NPoMs of the same volume, two new modes matching the triangular facet symmetry become important, (33) and (66), which better fit into each sharp apex, and with strongest intensities at the edges. We note (*lm*)_c_ consists of two modes with different radiation efficiency, which tune across each other (Figure 5c). Their interference does not contribute additional SERS variability since the same spread is seen when correlating SERS intensity with (*lm*)_b_ (Figure S10a, Supporting Information) which is a single mode.

The simulated far‐field emission patterns from NDoMs (Figure 5a) show that they all emit at high angles >50°, whereas only the (33) mode gives directional emission. The directional angle corresponds to the orientation of the NDoM apex on the mirror (bottom left Figure 5a). The characteristic horseshoe pattern of NDoMs then originates from interference between the near‐degenerate (33) and (20) modes. This high‐angle emission is confirmed in angle‐dependent measurements (Figure 5e; and Figure S17, Supporting Information). Weak near‐normal extra emission likely comes from the weaker “dark modes” (Figure 5b, gray stars).

The triangular facet strongly perturbs the original modes found under a circular facet. In particular, the dominant (20) NDoM mode has its strongest optical field now localized at the facet edges, rather than at the center as for NPoMs (Figure 5a). All the radiative NDoM modes have similarly localized fields at the facet edges, with predicted coupling strength estimated from the summed field around the facet perimeter $P\;=\int E.dl_{P}\;/\int \left|E\right|.dl_{P}$. For modes such as (22), which do not match the facet symmetry, both positive and negative contributions cancel out, whereas the strong apex fields for (*n*0) and (3*n*, 3*n*) with *n* an integer, give large *P*. Unequal field leakage from the facet edges onto the sides and top of the nanoparticle faces thus enhances outcoupling for *z*‐dipoles in the gap (produced in scattering). For the circular facet nanoparticles, the (3*n*, 3*n*) mode fields cancel around the perimeter, and these modes have negligible outcoupling.

We note one further consequence of precisely defining the Au(111) crystal plane of NDoM facets. This is controlling the probability of light‐induced atomic‐scale deformations that further localize the optical field within the nanocavity. When tracking the SERS from nanocavities, two types of transient spectral features have been identified that can suddenly appear: a) sets of energy‐shifted vibrational lines from single Au adatoms interacting with a single molecule (“picocavities”)^[^ ^9^, ^10^, ^24^ ^]^ and b) a broad‐spectrum background emitted from the top monolayer of Au atoms (“flares”).^[^ ^38^ ^]^ At average incident intensities of 50 µW µm^−2^, we find almost exclusively picocavities in NPoMs, and almost exclusively flares in NDoMs (Figure S18, Supporting Information). This suggests generation of picocavities and flares are strongly affected by the top facet, although with a different molecule picocavities mostly emerge from the NPoM mirror.^[^ ^39^ ^]^ While the (100) facet of cubic nanoparticles shows similar suppression of picocavities,^[^ ^40^ ^]^ its origin is not yet clear, and under further study. We speculate here that single crystal facets can better support large‐scale Au monolayer deformations, perhaps due to better organization of the bound surface molecular layer, although no differences in their SERS spectra can be identified. It may also be due to stronger field enhancements around the facet edges of NDoMs as compared to NPoMs, thus set by the facet shape (rather than facet crystal plane). In any case, this demonstrates the vital need to control optical nanocavities at the atomic level of morphology, and the utility of NDoMs for achieving this.

### 2.6 NDoM Nanocavity Elaboration

**Figure 6:**
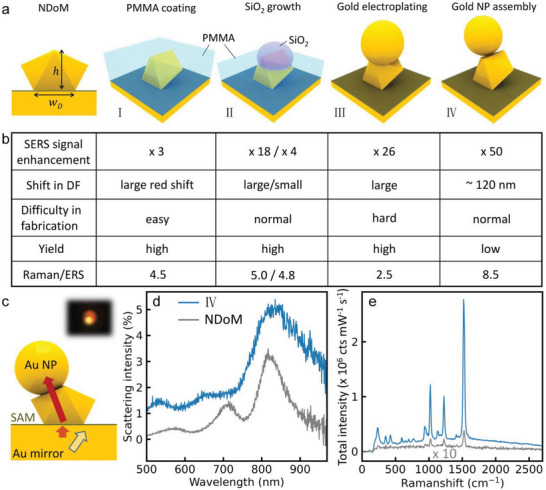
Creating high radiative efficiency nanoantennas from elaborated NDoMs. a) Schematics of NDoM and proposed geometries I‐IV that enhance light in/out coupling. In I, NP is fully embedded in PMMA while in II etched back PMMA is below NP top. b) Performance summary of the proposed geometries in (a), giving average values. Large indicates a shift > 100 nm. c) Geometry IV side view and out‐coupling of dipoles, inset: dark field image. d,e) Scattering and SERS spectra of geometry IV (blue) and NDoMs (gray).

Better defined optical confinement at the nanoscale can be utilized in many devices. However, while decahedra give much more precise nanocavities (especially compared to dimers of typically faceted AuNPs),^[^ ^41^ ^]^ and have nearly 100% higher overall SERS (for 633 nm pumping), they have 4 times weaker SERS per facet molecule. This is because their average height is closer to the Au mirror, and thus their antenna coupling to free space is lower than in NPoMs (**Figure** **6**a, left). To show how this can also be tackled, we adopt a sequential elaboration (Figure 6a I‐IV) that further demonstrates the robustness of the self‐assembled architectures employed.

Embedding NDoMs in a dielectric layer is the most straightforward method and gives a large increase of light in‐coupling for NPoMs.^[^ ^42^ ^]^ Here, an 80 nm poly(methyl methacrylate) (PMMA) layer is spin‐coated onto the substrate to fully cover all NDoMs (Figure 6a I). All NDoMs of each size show exactly the same average threefold SERS enhancement for excitation wavelengths of both 633 and 785 nm (see Note S1, Supporting Information). At the same time, modes in the DF spectra experience large redshifts ≈ 100 nm and become barely visible (Figure S17b,f, Supporting Information), although the transverse mode intensity increases. We suspect high angle emission is totally internally reflected at the PMMA‐air interface.

In geometry II, we selectively grow a 30–40 nm SiO_2_ nanosphere onto each NDoM by first exposing the upper NDoM Au surface with O_2_ plasma etching (see Note S2, Supporting Information), before nucleation and growth using a silane coupling agent.^[^ ^43^ ^]^ Such SiO_2_ spheres have been found to act as nanolenses that improve both in/out coupling of the nanocavity.^[^ ^43^ ^]^ Indeed, a much higher 18‐fold enhancement of SERS (Figure S21, Supporting Information) is recorded, but after dissolving the PMMA film it drops back to fourfold improvement. All cavity modes can however be clearly identified in the DF spectra of these NDoMs+SiO_2_ nanosphere (Figure S21c, Supporting Information). The enhancement could be further increased by enlarging the size of the SiO_2_ spheres, which however would complicate the DF spectra.^[^ ^43^ ^]^ While the facet is nicely controlled in this geometry, broadening of the SERS intensity distribution is seen for SiO_2_ growth on top. This can be attributed to the inhomogeneous growth of SiO_2_, which could be solved by further optimization.

To avoid the interfacial complexity of such metallo‐dielectric constructs, in situ electrochemical deposition can also be used to increase the NDoM height without changing the bottom facet (geometry III). To localize growth solely on the NDoM top, a protective PMMA layer is spin‐coated onto the sample and etched back to reveal the top NDoM facets (see Note S3, Supporting Information).^[^ ^44^ ^]^ Pulsed deposition is then used to grow additional Au of different volume (Figure S22, Supporting Information) by tuning the deposition time, after which the PMMA is dissolved. DF images show the deposition is highly uniform at a large scale. The DF scattering increases strongly by >10‐fold (Figure S23, Supporting Information) with 5–10 s deposition times, while the SERS excited by 633 and 785 nm increases by >5‐fold and >25‐fold, respectively (Figure S23d, Supporting Information), exceeding that of NPoMs with the same volume. Full independent tuning of the antenna height and facet is thus possible, and can be arranged to enhance signals in any desired region of the spectrum. The modes in DF become less clear however, likely because the nanostructures reach 300 nm laterally, even with 5 s deposition time, suggesting further optimization of this deposition would be advantageous.

One extra strategy explored attaches spherical NPs selectively to the top surface of the PMMA‐protected decahedra (geometry IV), to more consistently increase the NDoM height and further break the symmetry of the nanoantenna (see Note S4, Supporting Information). The DF image of geometry IV (Figure 6c inset) shows additional symmetry breaking, with a bright spot now on one side of the horseshoe pattern. The DF spectra only increase in intensity by 70% (Figure 6d) due to the involving of an extra Au NP of similar volume to the nanodecahedron. Also, a significant broadening of the cavity modes compared to NDoMs is observed, but the modes remain highly consistent (Figure S25g, Supporting Information). Most importantly, a near 100‐fold SERS enhancement (Figure 6e, >50‐fold on average) is obtained (Note S4, Supporting Information), now two orders of magnitude more than NPoMs. In addition, the SERS intensity increases several times more than the SERS backgrounds, which suggests the penetration of light into the metal is reduced. This result implies the strategy of retaining a consistent facet while increasing the height of the nanoantenna works well for increasing light in/out coupling. The enhancement can be attributed to two factors: increased coupling efficiency to free space, and lower angle emission (Figure 6c inset) that increases the collection efficiency. While this tradeoff is still to be quantified, our work demonstrates a highly promising geometry that solves many technical issues for plasmonic nanocavities.

A comprehensive comparison of these elaborated geometries (Figure 6b) summarizes the trade‐offs. Geometry I is the simplest method to increase light out‐coupling in terms of fabrication. Although its enhancement is smaller, the method is universal and highly adaptable for any nanocavity structure. While a limited enhancement is found for SiO_2_ nanolenses alone, the hybrid SiO_2_ + PMMA coating (geometry II) provides ≈ 20‐fold larger enhancements. Nevertheless, because scattering spectra become more blurred, it gives a less precise understanding. Geometry III supports a large signal enhancement and tunable cavity modes without changing the facet or gap size. The Au light emission strongly increases along with the SERS (likely because the deposited Au has crevices) but may be further optimized by reducing the volume of deposited metal. The highest enhancements and ratio of SERS:Background are obtained in geometry IV, as well as consistent DF spectra. Combinations of these approaches may also be fruitful, for instance geometries I+III should yield enhancements exceeding 100. However, the yield of fabricating such geometries is so far low (see Note S4, Supporting Information), and a focus of further work. In general however, all the geometries here are reproducible sequential elaborations retaining the atomically‐precise nanogaps, which opens a wide range of nanocavity exploitation paths.

## 3 Conclusions

We showed that plasmonic nanocavities of few nm thickness are exquisitely sensitive to facet size and shape. Typical spherical nanoparticles have poorly‐controlled faceting, so that, even in the nanoparticle‐on‐mirror geometry where facet registration is not an issue (compared to dimers), the cavity modes and field enhancements vary. By measuring more than 20 000 such constructs, we showed that the decahedron‐on‐mirror (NDoM) configuration gives much improved performance due to its precise facet shape and crystal plane. We find that, although decahedra are canted, most modes radiate symmetrically to high angles from a *z*‐oriented dipole, apart from one mode whose field is strongest toward the central 5‐vertex. Optical images can thus ascertain the NDoM orientation, allowing additional control of relative polarization during illumination. We explore all possible structural‐related spectroscopic measures of the NDoMs to locate a high correlation between SERS intensity (near field) and elastic scattering (far field). However, improvements from polarization‐sensitive spectroscopy suggest that small chiral facet variations remain to be easily identified.

The precise effects on SERS can be better understood from a deeper understanding of how cavity modes are excited. By synthesizing a wide range of NDoM sizes, we demonstrate tuning over the visible and NIR spectral regions, and show resonant tuning to different Raman pump lasers. Strong but consistent SERS (and all other nonlinear processes) in these nanocavities can be supplemented by higher in/out‐coupling using multistage elaboration of the antenna component of NDoMs. We successfully create a highly efficient nanocavity giving average SERS intensities 100‐fold stronger than normal NPoMs. A range of atom‐sensitive phenomena can also now be explored including light‐induced van‐der‐Waals interactions that create picocavities and flares, as well as molecule‐metal interactions in molecular electronics, quantum emitters, (photo)catalysis, electrochemistry, and (bio)sensing.

## 4 Experimental Section

### Chemicals

Gold (III) chloride trihydrate (HAuCl_4_, ≥ 99.9%), hexadecyltrimethylammonium chloride (CTAC, 25% in water), citric acid (99.5%), sodium borohydride (NaBH_4_, 99%), benzyldimethylhexadecyl‐ammonium chloride (BDAC), ascorbic acid (AA, ≥ 99%), poly (sodium 4‐styrenesulfonate) (Na‐PSS, *M*_w_ = 70 000 g mol^−1^), sodium citrate tribasic dihydrate (≥ 99%), BPT (97%), and ethanol (99.5%) were purchased from Aldrich‐Merck. All chemicals were used without further purification. Milli‐Q water (resistivity 18.2 MΩ cm at 25 °C) was used in all experiments. All glassware and stir bars were washed with aqua regia and rinsed with Milli‐Q water prior to use.

### Synthesis of Gold Decahedra

Gold decahedra were synthesized according to a published procedure using seed‐mediated growth.^[^ ^28^ ^]^ Gold seeds were first prepared by fast reduction of HAuCl_4_ with NaBH_4_ in the presence of both a cationic surfactant (CTAC) and citric acid in a 20 mL scintillation vial. To an aqueous mixture containing CTAC (10 mL, 50 mm), HAuCl_4_ (0.05 mL, 50 mm), and citric acid (0.05 mL, 1 m), freshly prepared NaBH_4_ (0.25 mL, 25 mm) was added under vigorous stirring at 20 °C. The solution color turned from yellow to brownish immediately. Two minutes later, the seed solution was aged by closing the vial with a screw cap and heating at 80 °C for 90 min in a silicone oil bath, under gentle stirring. During aging, the solution color turned gradually from brown to red, indicating an increase in nanoparticle size. Finally, the solution was removed from the bath, stored at room temperature, and used without further treatment. Gold decahedra were prepared by adding a desired volume of gold seeds (1.42, 1.0, and 0.34 mL to obtain gold decahedra with an edge length of 22, 30, and 41 nm, respectively) to a growth solution containing BDAC (50 mL, 100 mm), HAuCl_4_ (0.5 mL, 50 mm), and AA (0.375 mL, 100 mm) at 30 °C, under vigorous stirring. The mixture was then left undisturbed at 30 °C for 30 min.

To grow decahedra of larger edge length, gold decahedra with an edge length of 41 nm were used as seeds. Gold decahedra with an edge length of 51, 70, and, 81 nm were synthesized by adding a specific volume of gold decahedra seeds (2.4, 0.7, and 0.4 mL, [Au^0^] = 5 mm) to a growth solution containing BDAC (50 mL, 15 mm), HAuCl_4_ (0.25 mL, 50 mm), and AA (0.19 mL, 100 mm) at 30 °C, under vigorous stirring for 60 min. The gold decahedra dispersions were centrifuged twice (7000–3000 rpm, 30 min) to remove excess reactants and dispersed in aqueous CTAB solution (10 mm). The final concentration of metallic gold was 0.4 mm.

### Citrate Functionalization

A colloidal dispersion of gold decahedra (50 mL, 0.4 mm) in CTAB (10 mm) was subjected to three cycles of centrifugation and redispersion with Na‐PSS (50 mL, 0.15% wt) to remove CTAB. The PSS‐stabilized gold decahedra were centrifuged and redispersed in sodium citrate (50 mL, 5 mm) and the solution was incubated overnight to exchange PSS. Finally, gold decahedra were subjected to two additional centrifugation and redispersion cycles using sodium citrate (50 mL, 5 mm), yielding stable dispersions of citrate‐stabilized gold decahedra.^[^ ^45^ ^]^

### Nanoparticle Characterization

TEM images were acquired on a JEM‐1400 microscope operating at 120 kV. Optical extinction spectra were recorded using an Agilent 8453 UV–visible spectrophotometer. The dimensions of the obtained gold decahedra were determined by TEM image analysis, by measuring more than 100 randomly chosen nanoparticles.

### Sample Preparation

Spherical gold nanoparticles of 80 and 60 nm diameter stabilized with citrate were purchased from BBI Solutions. Ultrasmooth gold substrates were fabricated by the template‐stripping method.^[^ ^46^ ^]^ The self‐assembled monolayers (SAMs) of BPT molecules were assembled by immersing the gold substrates into a 1 mm BPT solution for 16 h. Afterward, the substrates were rinsed with a large amount of ethanol to remove the physically absorbed molecules and dried with nitrogen. NPoMs and NDoMs were then fabricated by drop‐casting the nanoparticles on top of the SAMs for 10 s. Finally, the substrates were rinsed with ultrapure water and dried with nitrogen. The procedures of fabricating the elaboration geometries are comprehensively described in Notes S1–S4 (Supporting Information).

### Single Nanoparticle DF and SERS Measurements

The SERS and DF spectra were obtained on a home‐built confocal Raman microscope by splitting the signal into two channels and reading out with Andor EMCCD and Ocean Optic spectrometer, respectively. Single nanoparticle spectroscopy was enabled by limiting the slit width and the readout lines on the CCD to isolate optical signals from a particle. DF images were acquired with a Lumenera Infinity2 camera. The 633 and 785 nm single‐frequency diode laser beams were coupled into the microscope and aligned to focus on the same area of the sample for taking two wavelengths SERS measurements. Laser powers were measured after passing through the microscope objective using a Thorlabs PM16‐121 power meter. The SERS intensity of different excitation lasers was normalized using the Raman signal from undoped silicon wafers (520.6 cm^−1^) to give the instrument response. DF spectra were normalized with a Labsphere Spectralon reflectance standard to correct for efficiency of both collection and light source. An Olympus DF objective of NA0.9 was used for all measurements to ensure high efficiency collection of high angle radiation. Automatic particle tracking was carried out using an in‐house algorithm integrated into python that coordinates all the computer‐controlled instruments including motorized sample stage, spectrometer, lasers, and acousto‐optic modulator. For each nanocavity, an automatic scan through the focal depth is performed to record DF spectrum with integration times of 1s. The depth‐dependent intensity at each wavelength was fitted with a Gaussian shape to extract the chromatic aberration corrected scattering spectrum (see Figure S1, Supporting Information).

### COMSOL Simulation

Simulations were performed with COMSOL using a Finite Element Method (FEM) with the adaptation of the QNMEig toolkit.^[^ ^25^ ^]^ Parameters such as SAM reflective index and dielectric constant of Au were set according to previous simulations.^[^ ^47^ ^]^ The simulated NDoM geometry had a gap size of 1.5 and 5 nm bottom facet rounding.

## Conflict of Interest

The authors declare no conflict of interest.

## Supporting information

Supporting Information

## Data Availability

The data that support the findings of this study are available from the corresponding author upon reasonable request.
