# Supplementary material for: Full Control of Plasmonic Nanocavities Using Gold Decahedra‐on‐Mirror Constructs with Monodisperse Facets
Source: Adv Sci (Weinh). 2023 Feb 3;10(11):2207178. doi: 10.1002/advs.202207178 (PMC10104671; doi:10.1002/advs.202207178)
Supplement: Supplementary file 1 — Supporting Information [file ADVS-10-2207178-s001.pdf]

ADVANCED SCIENCE
Open Access

Supporting Information

for *Adv. Sci.*, DOI 10.1002/advs.202207178

Full Control of Plasmonic Nanocavities Using Gold Decahedra-on-Mirror Constructs with Monodisperse Facets

*Shu Hu, Eoin Elliott, Ana Sánchez-Iglesias, Junyang Huang, Chenyang Guo, Yidong Hou, Marlous Kamp, Eric S. A. Goerlitzer, Kalun Bedingfield, Bart de Nijs, Jialong Peng, Angela Demetriadou, Luis M. Liz-Marzán and Jeremy J. Baumberg**

Supplementary Information

Full control of plasmonic nanocavities using gold decahedra-on-mirror constructs with monodisperse facets

Shu Hu[1], Eoin Elliott[1], Ana Sánchez-Iglesias[2], Junyang Huang[1], Chenyang Guo[1], Yidong Hou[1], Marlous Kamp[1], Eric S. A. Goerlitzer[1], Kalun Bedingfield[4], Bart De Nijs[1], Jialong Peng[1], Angela Demetriadou[4], Luis M. Liz-Marzán[2,3], Jeremy J. Baumberg[1*]

[1] Nanophotonics Centre, Department of Physics, Cavendish Laboratory, University of Cambridge, Cambridge, CB3 0HE, England, UK

[2] CIC biomaGUNE, Basque Research and Technology Alliance (BRTA), Paseo de Miramón 194, Donostia-San Sebastián 20014, Spain

[3] Ikerbasque, Basque Foundation for Science, Bilbao 43009, Spain

[4] Department of Physics and Astronomy, University College London, London WC1E 6BT, United Kingdom School of Physics and Astronomy, University of Birmingham, Edgbaston, Birmingham, B15 2TT, UK

* e-mail: jjb12@cam.ac.uk

SUPPLEMENTARY FIGURES

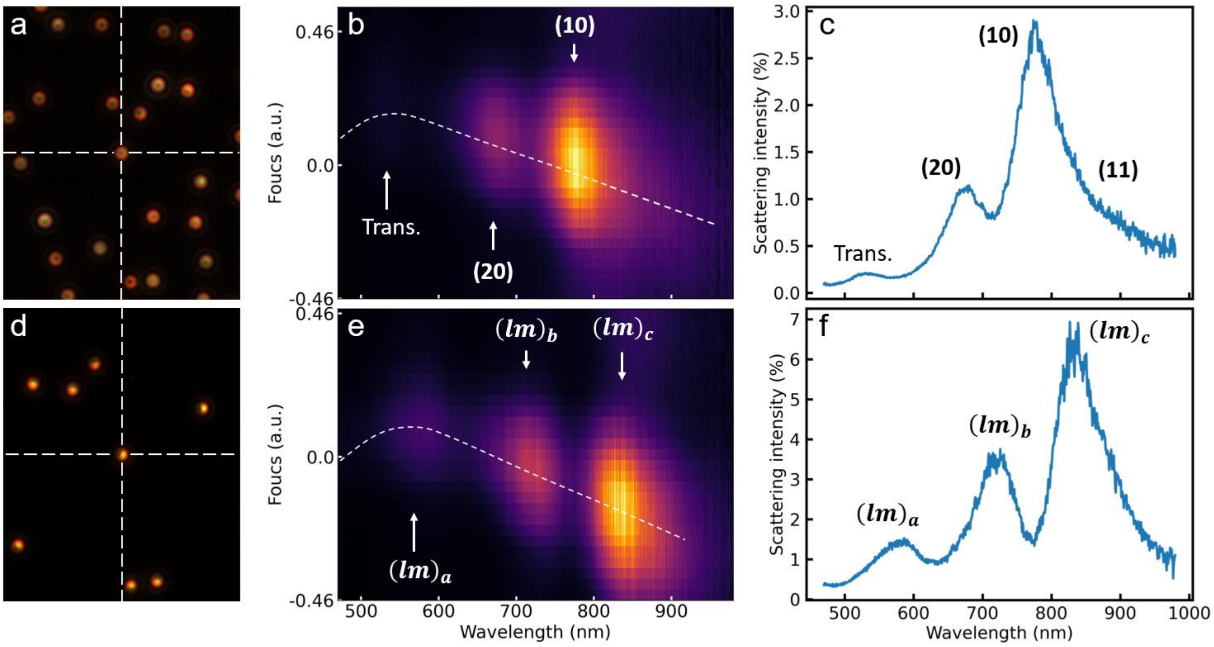

Supplementary Figure 1. Single nanoparticle DF spectral measurements of 80 nm NPoM and 81 nm NDoM. a,d DF images of (a) NPoMs and (d) NDoMs. Crossed dashed lines indicate signal collection spot. **b,e** Scattering spectra scans changing the focal height through the focus of single (b) NPoM and (e) NDoM nanocavities. The dashed lines show the chromatic dispersion of each wavelength at different focal distances. **c,f** Extracted scattering spectra of (c) single NPoM and (f) NDoM after correction of chromatic aberration.

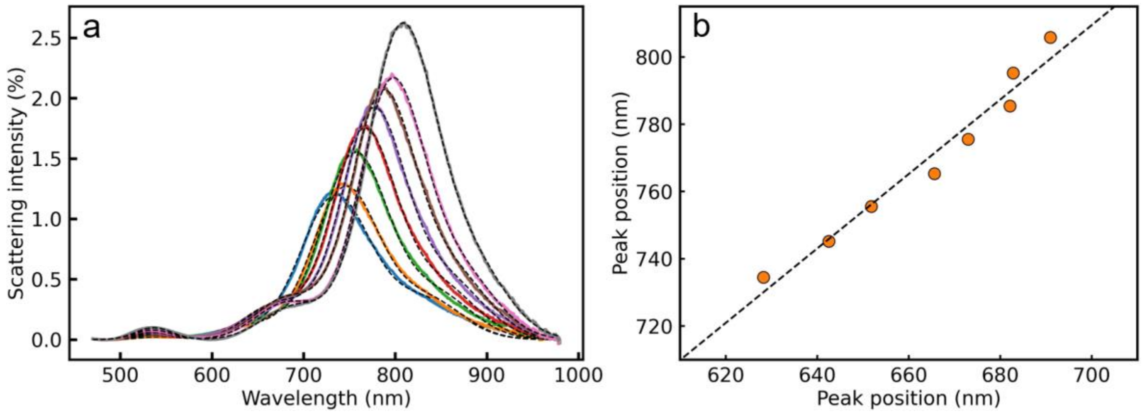

Supplementary Figure 2. Scattering spectra statistics of 80 nm NPoMs. (a) Average spectra (same spectra as in Figure 1b but with removal of scattering background) and their fitting curves (black dashed lines) in each bin of the histogram in Figure 1b. (b) Peak position correlation of (10) and (20) modes of the average spectra in (a).

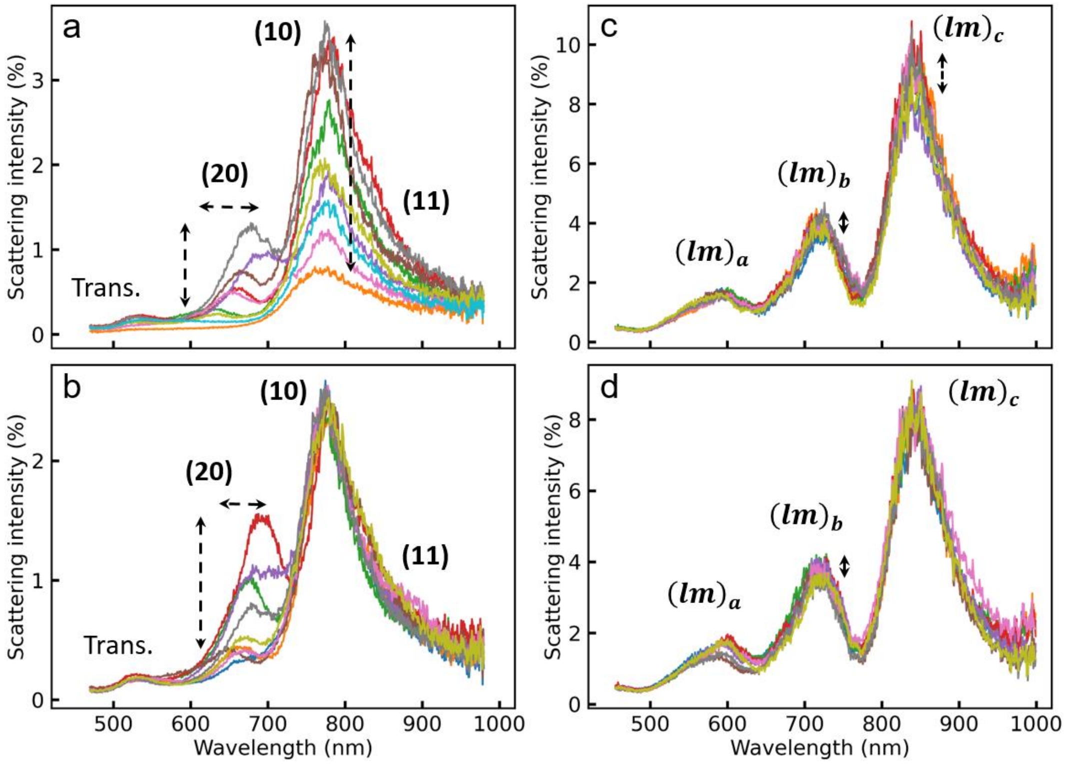

Supplementary Figure 3. Randomly selected single nanocavity spectra of 80nm NPoMs and 81nm NDoMs. The scattering spectra of individual (a-b) NPoMs and (c-d) NDoMs with dominant mode energy at the bin centre of the histograms from Figure 1 (b,d) with and (a,c) without constraining the scattering intensity.

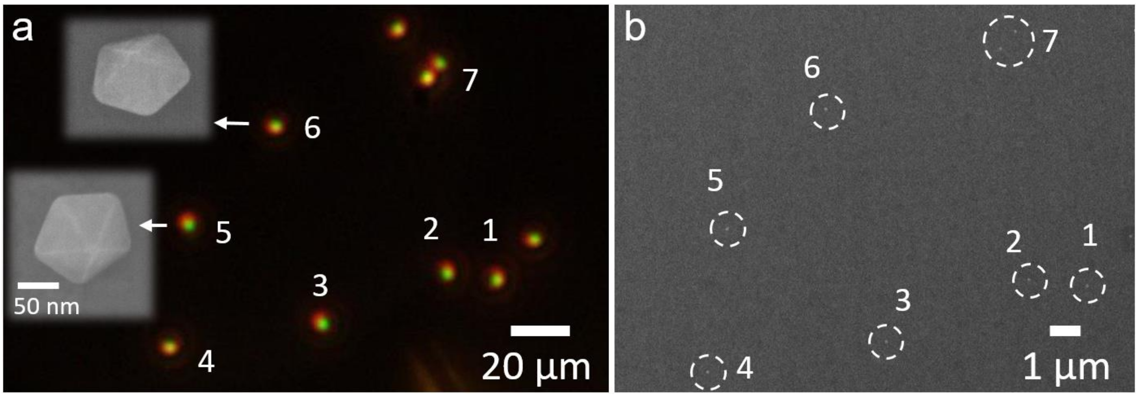

Supplementary Figure 4. (a) DF and (b) SEM co-localized measurements of the same NDoMs. The inset in (a) shows the correlation between the DF patterns and the orientations of the NDoMs.

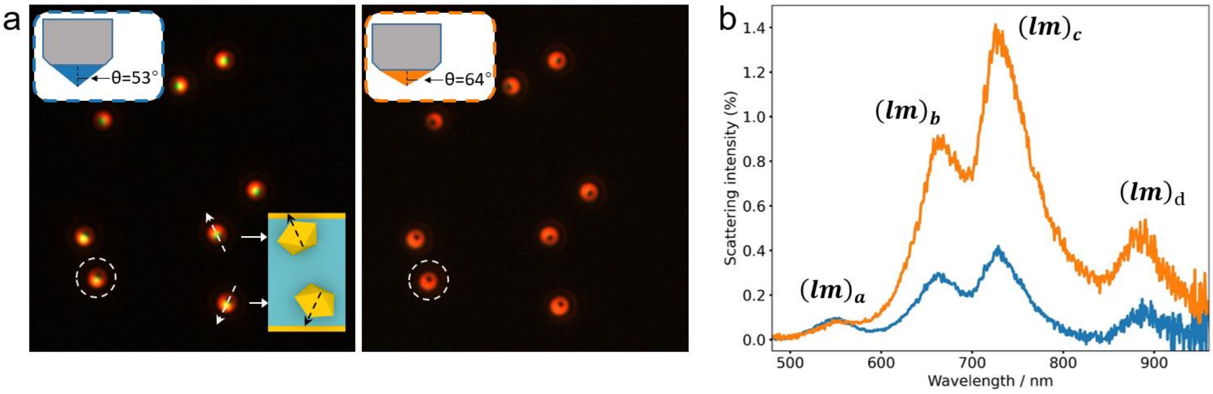

Supplementary Figure 5. Effects of numerical aperture. (a) DF images and (b) spectra of the same 51nm NDoMs recorded with objectives of different collection angle, showing high angle light dominates.

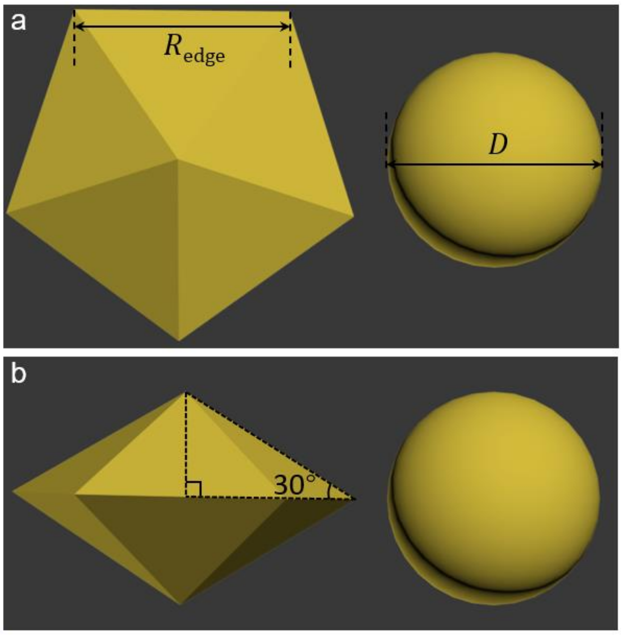

Supplementary Figure 6. (a) Top and (b) side views of Au decahedron and sphere with matching edge size and diameter. The volume of Au decahedron and sphere are $0.54\,(R_{\mathrm{edge}})^3$ and $0.52\,D^3$ respectively. Assuming equal volumes, $R_{\mathrm{edge}} = 0.99\,D$. The tip angle of such Au decahedra is 30° as measured in [ref [1]].

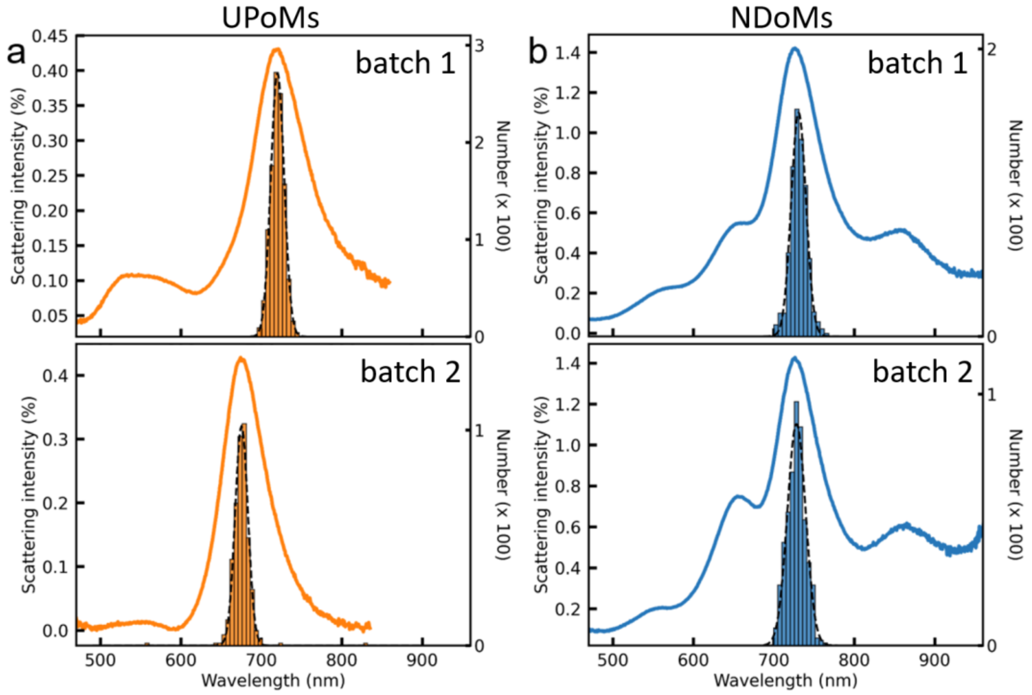

Supplementary Figure 7. DF spectra histograms of (**a**) UPoMs and (**b**) NDoMs, of different batches.

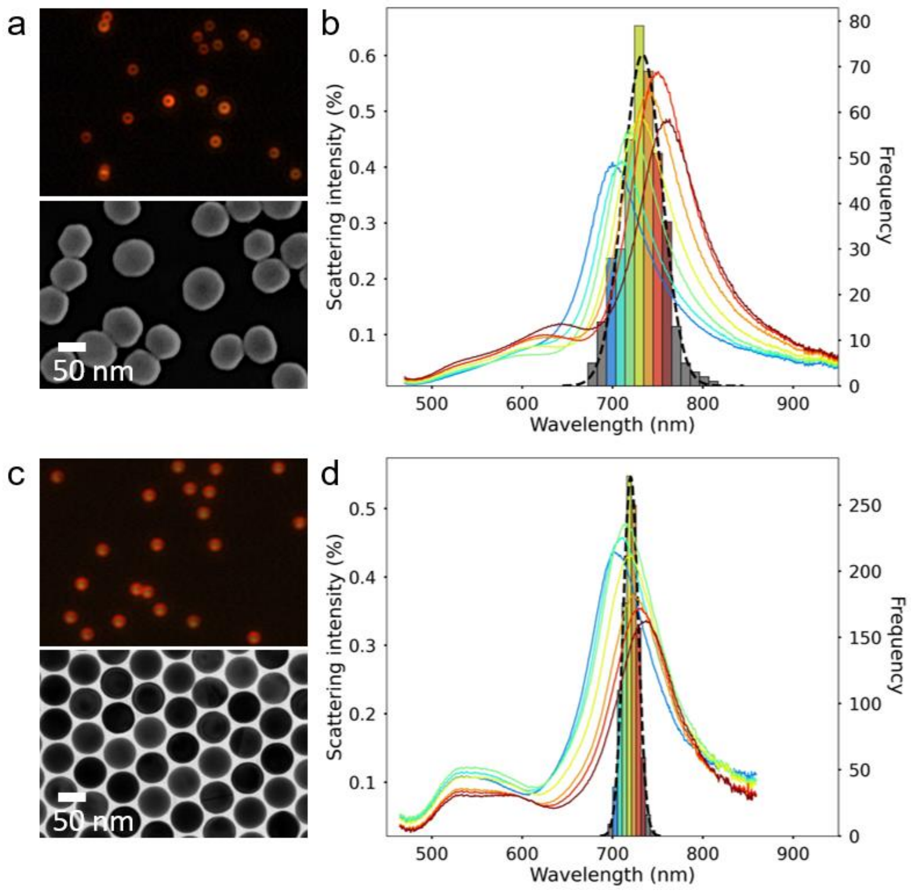

Supplementary Figure 8. Comparison of 60nm NPoMs fabricated with normal and ultraspherical Au NPs. (a,c) DF (top) and SEM (bottom) images of (a) normal and (c) ultraspherical Au NPs. (b,d) Histograms sorted by dominant mode wavelength of (b) normal and (d) ultraspherical Au NPs.

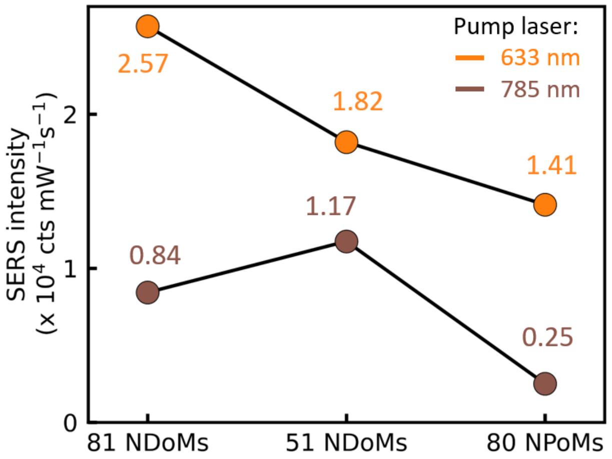

Supplementary Figure 9. SERS strength for different constructs. SERS peak heights from Gaussian fit of SERS intensity distributions of 81nm NDoMs, 51nm NDoMs and 80 NPoMs in Figure 2.

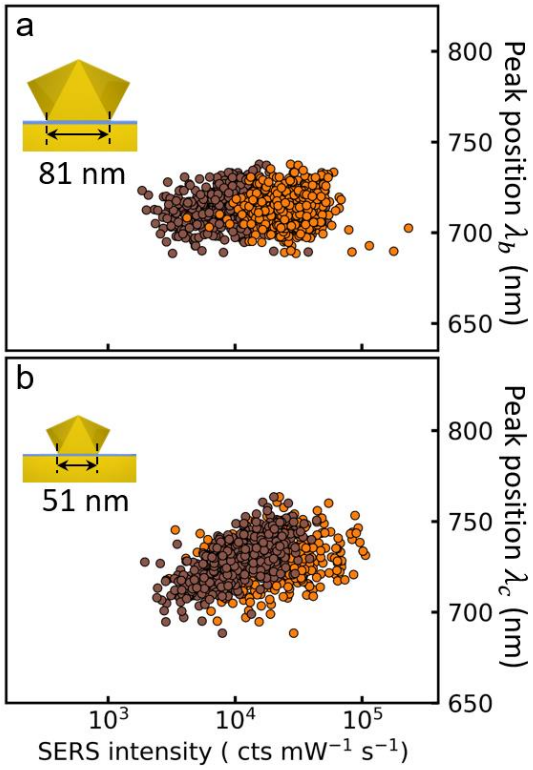

Supplementary Figure 10. Correlation of mode positions and SERS intensities. Correlation of (a) $(lm)_b$ and (b) $(lm)_c$ mode wavelength with 1550 cm^{-1} SERS intensity for (a) 81 nm and (b) 51 nm NDoMs. The orange and brown points are SERS excited with 633 nm and 785 nm lasers respectively.

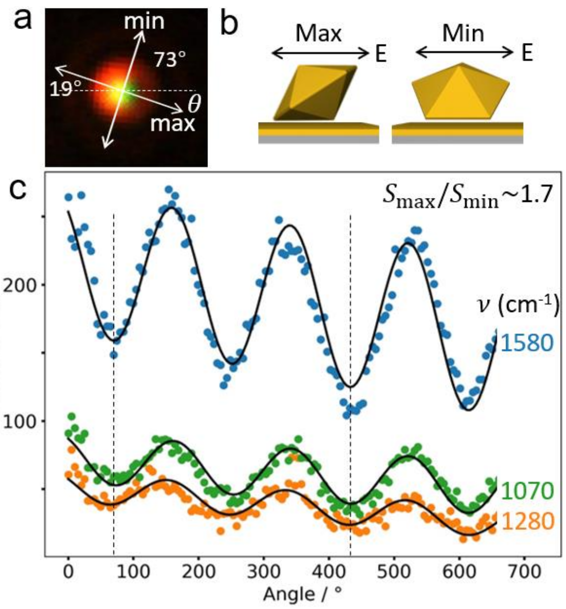

Supplementary Figure 11. Pump polarisation dependence of SERS. (a,b) DF image showing the alignment of central 5-vertex along θ=-19°. Light polarisation direction of 633nm pump laser marked for largest and smallest SERS emission. (c) SERS measurements as laser linear polarisation is rotated.

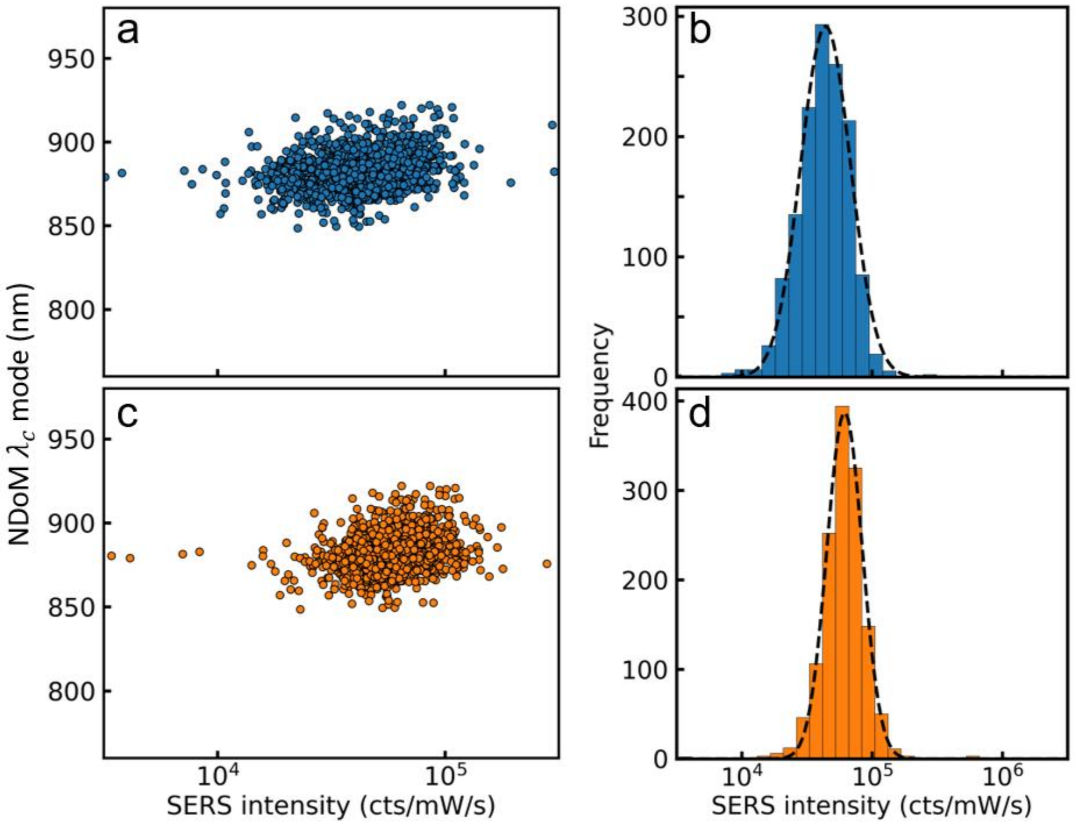

Supplementary Figure 12. Correlation of mode at SERS. (a, c) SERS vs $(lm)_c$ mode correlation and (b, d) SERS intensity distribution (a, b) before and (c, d) after correction of laser polarization.

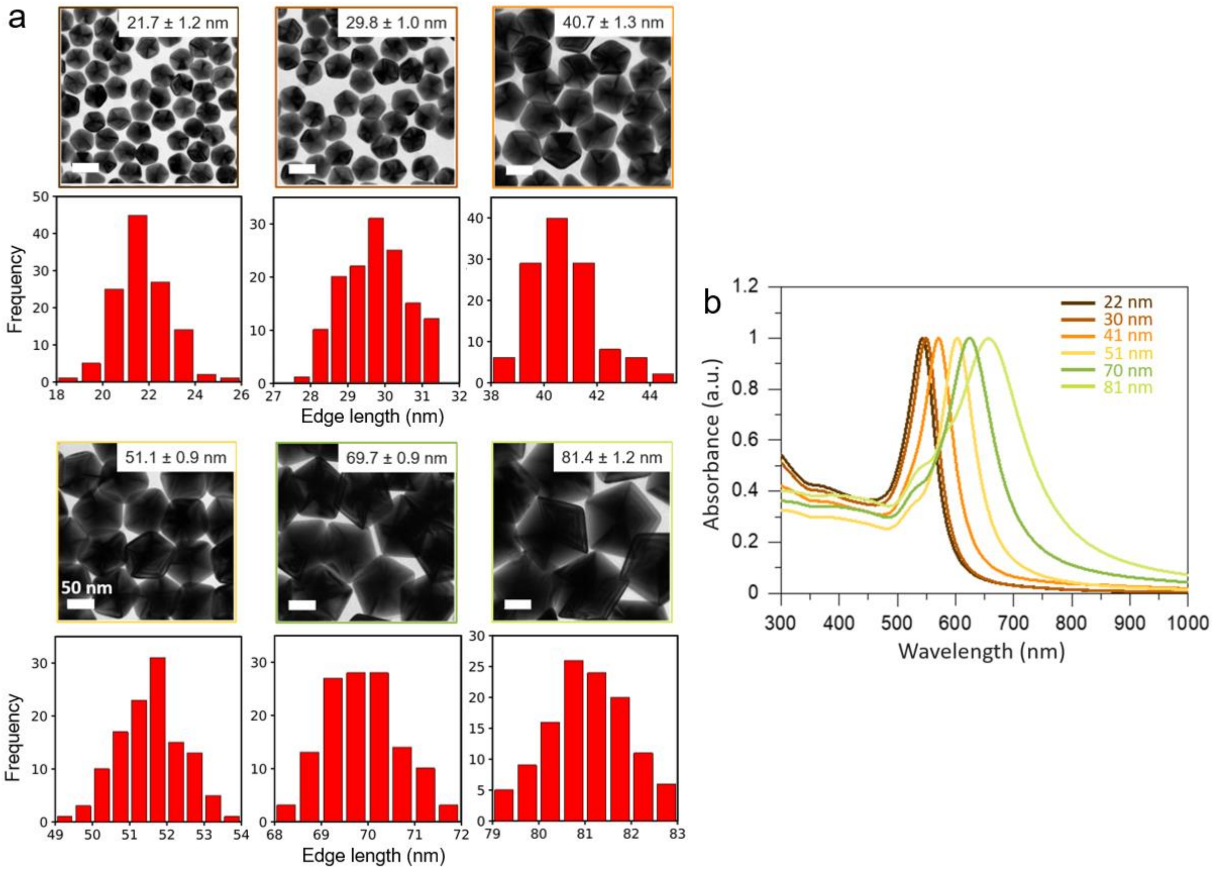

Supplementary Figure 13. Size-scaled decahedra. (a) TEM images, edge size distributions and (b) UV-vis extinction spectra of Au decahedra of increasing size. Insets in (a) show statistics of Au decahedron size over a large number of nanoparticles. The TEM images are also used in Figure 4a.

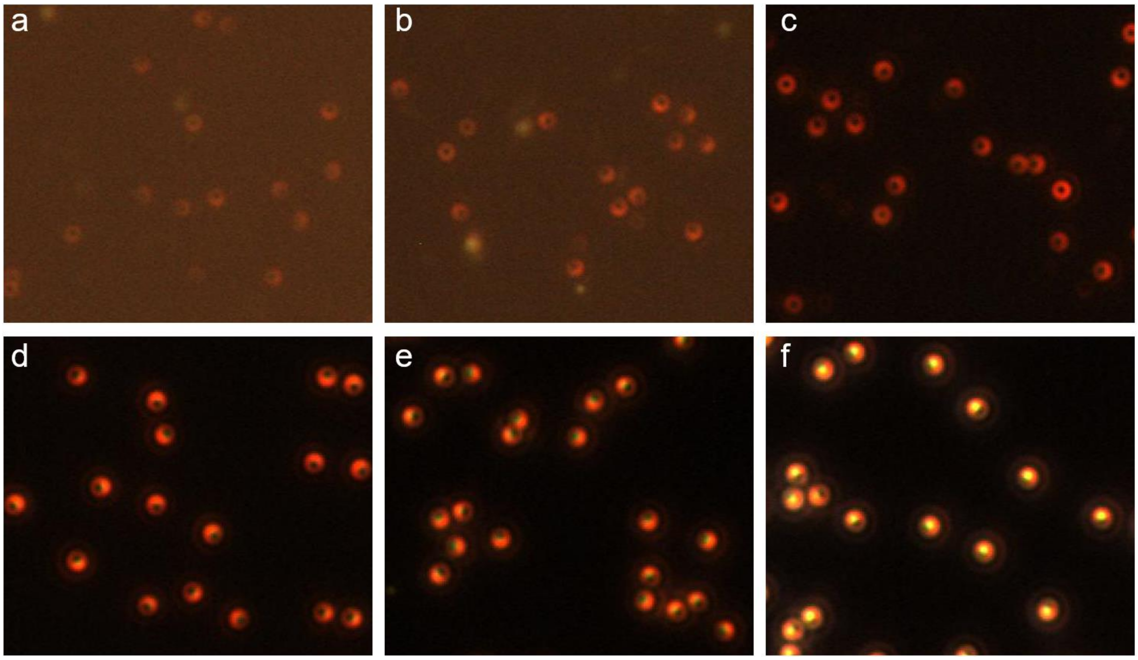

Supplementary Figure 14. DF images of NDoMs. Different NDoM sizes of (a) 22nm, (b) 30nm, (c) 41nm, (d) 51nm, (e) 70nm, and (f) 81nm.

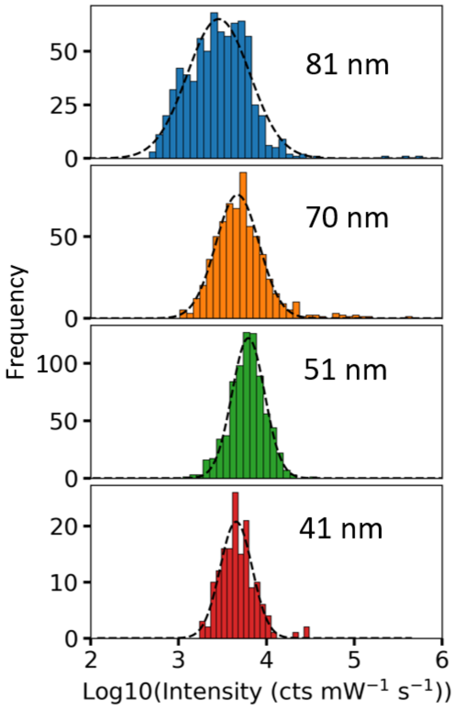

Supplementary Figure 15. The SERS intensity (1550 cm^{-1}) distributions for NDoMs with different sizes. (excitation laser: 785nm)

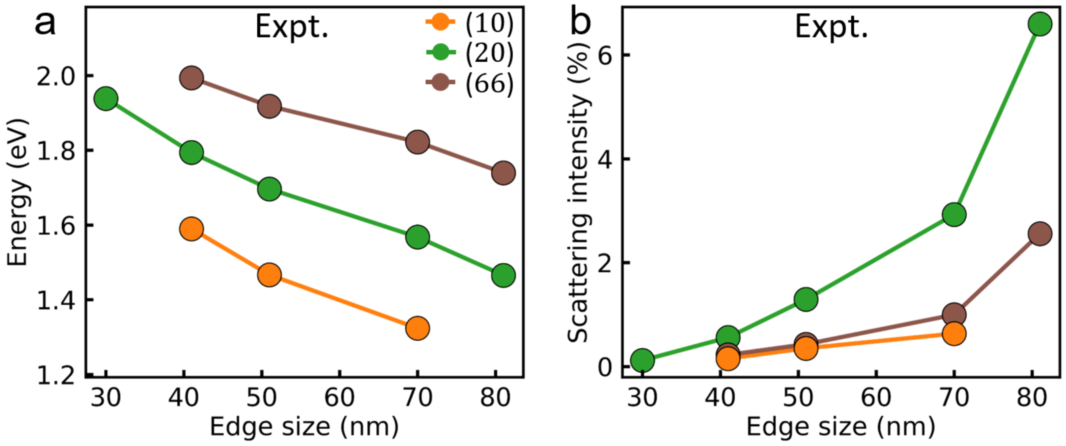

Supplementary Figure 16. Experimental size-dependent (**a**) average mode energy and (**b**) average scattering intensity of main NDoM cavity modes.

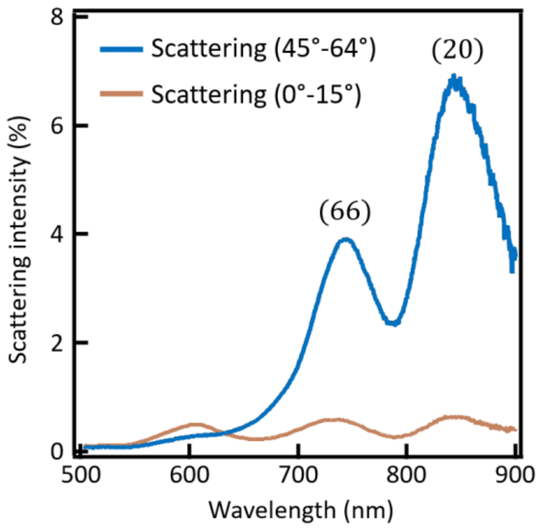

Supplementary Figure 17. Angular emission. High (45°-64°) and low (0°-15°) angle scattering spectra of a 81nm NDoM extracted from angular emission images.

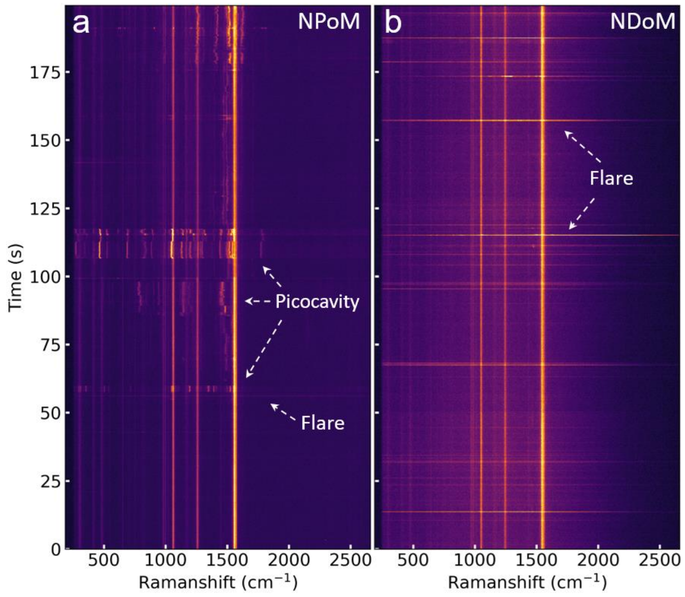

Supplementary Figure 18. Time-dependent dynamics of SERS spectra for 81nm NDoM and 80nm NPoM. Time-dependent SERS scans of (a) NPoM and (b) NDoM (excitation laser: 633 nm laser) recorded with the same laser power and exposure of 50µW and 0.2s respectively.

Supplementary Note 1. Fabrication and characterization of PMMA-coated NDoMs

To make a PMMA film, 50 µL PMMA (2% in anisole solution) was spin-coated onto NDoM substrates with a rotation speed of 3000 rpm. The substrates were then annealed on a hot plate at a temperature of 50 °C for 2 mins. The thickness of the PMMA film was measured by ellipsometry and etched back by O_2 plasma etching. In geometry I, the PMMA thickness is set to be 80 nm for fully covering the NDoMs of different sizes. Uncoated NDoM samples prepared in parallel were used as controls for SERS signal comparison. Extra SERS signal enhancements are induced by PMMA of 3 times irrespective of size and excitation wavelength (Supplementary Figs. 19, 20). The SERS intensity distribution width is not changed by the PMMA. The horseshoe pattern of the NDoMs turns into a single spot with the PMMA coating (Supplementary Figs. 19a,e), with the cavity modes now barely visible in scattering spectra (Supplementary Figs. 19b,f).

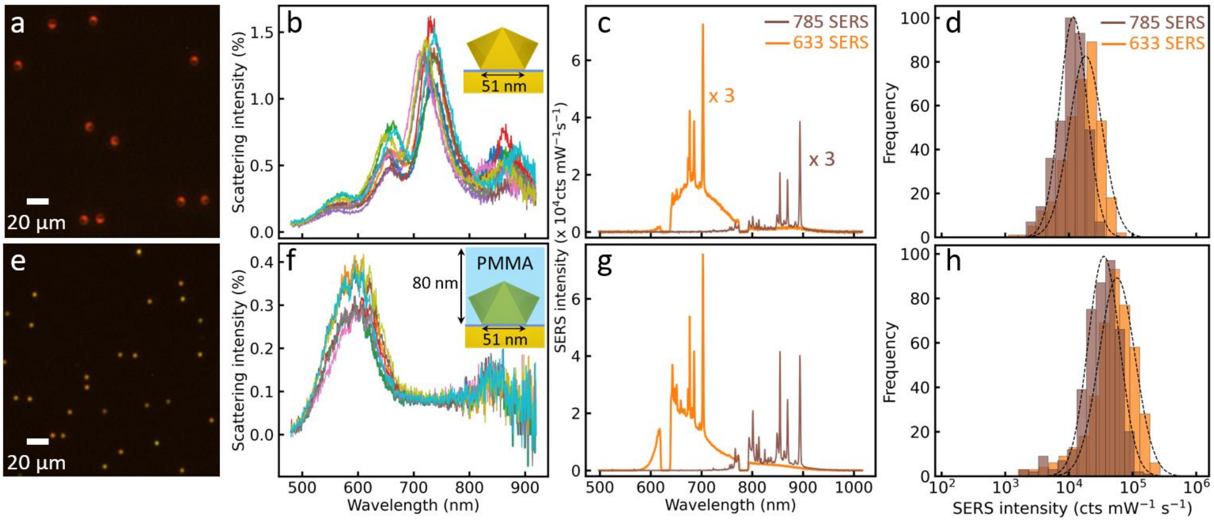

Supplementary Figure 19. DF and SERS characterization of PMMA-coated 51 nm NDoMs. DF (a,e) images and (b,f) spectra for 51 nm NDoMs (a,b) without and (e,f) with 80 nm PMMA coating. (c,g) Average SERS spectra and (d,h) SERS intensity distribution for 51 nm NDoMs (c,d) without and (g,h) with 80 nm PMMA coating.

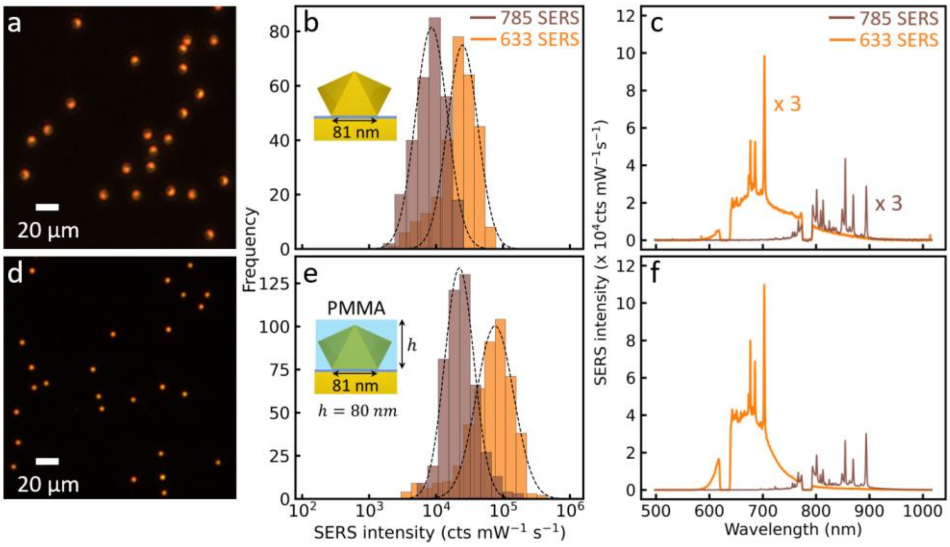

Supplementary Figure 20. DF and SERS characterization of PMMA-coated 81 nm NDoMs. DF images for 81 nm NDoMs (a) without and (d) with 80 nm PMMA coating. (b,e) SERS intensity distribution and (c,f) average SERS spectra for 81 nm NDoMs (b,c) without and (e,f) with 80 nm PMMA coating.

Supplementary Note 2. Fabrication and characterization of NDoMs with SiO_2 nanolenses

A wet-chemical method is applied to grow the SiO_2 sphere on top of NDoMs according to our previous work[2]. To avoid the SiO_2 growing in areas without NDoMs, PMMA is spin-coated onto the substrate and etched back by O_2 plasma (same procedures as Supplementary Note 1) for exposing the Au surface on the NDoM top. The SERS signal is enhanced around 3-fold after PMMA etching back to 40 nm (Supplementary Figs. 21a,b, blue and orange), which is consistent with PMMA-coated NDoM results. A large signal enhancement of 18-fold is observed when the SiO_2 is deposited on top (Supplementary Fig. 21b, green), while dropping back to 4-fold when the PMMA is stripped off by acetone. The DF spectra of NDoMs show a small shift before and after the SiO_2 is grown on top (Supplementary Fig. 21c), indicating the volume of SiO_2 is small.

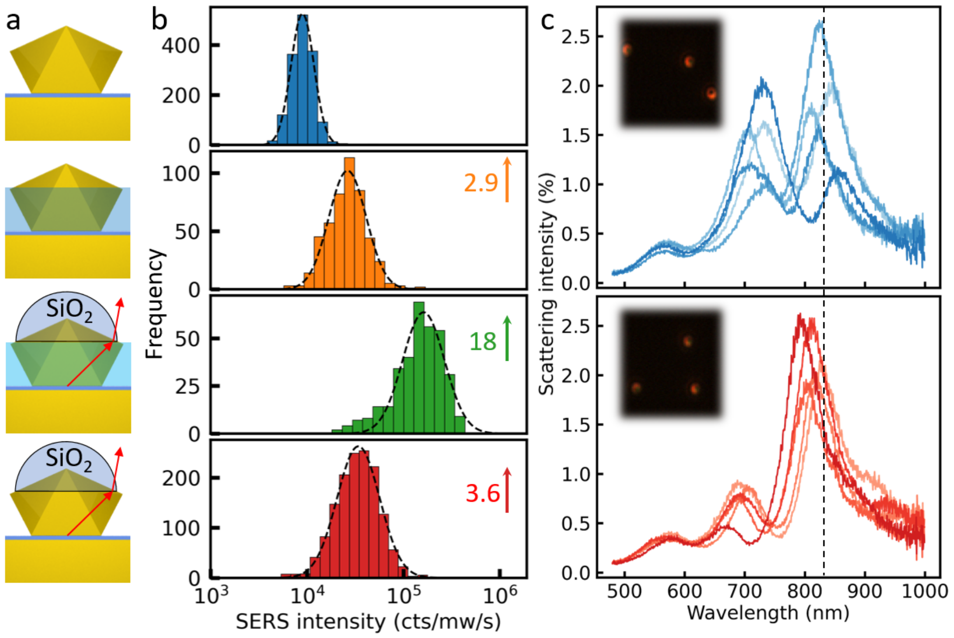

Supplementary Figure 21. The effect of SiO_2 nanolens on NDoMs. (a) Schematics and (b) SERS intensity distribution of four different geometries. (c) DF images and spectra comparing the first (blue) and last (red) geometries.

Supplementary Note 3. NDoM elaboration using electrochemical deposition

As in Supplementary Note 2, we spin-coated a PMMA layer (90 nm) on top of the NDoMs (Supplementary Fig. 22a) and etched it back to expose the Au surface (the final thickness of PMMA is 35 nm). The substrate is then placed into an electrochemical cell under potential control. To minimize the surface roughness, pulse potential deposition is performed , with the potential held at E_1 for 200 ms to trigger deposition and E_0 for 800 ms to equilibrate the diffusion (Supplementary Fig. 22g)[3]. The PMMA is stripped off by rinsing with acetone after deposition. A significant scattering intensity increase is observed for each NDoM after the Au deposition while the rest of the substrate remains clean (Supplementary Figs. 22b-e). This method allows uniform Au deposition over a large time range (Supplementary Figs. 22h,i) of high reproducibility. Deposition times of 5 s create an identical spherical Au nanostructure of ~ 300 nm diameter on top of each NDoM (see Supplementary Fig. 22f).

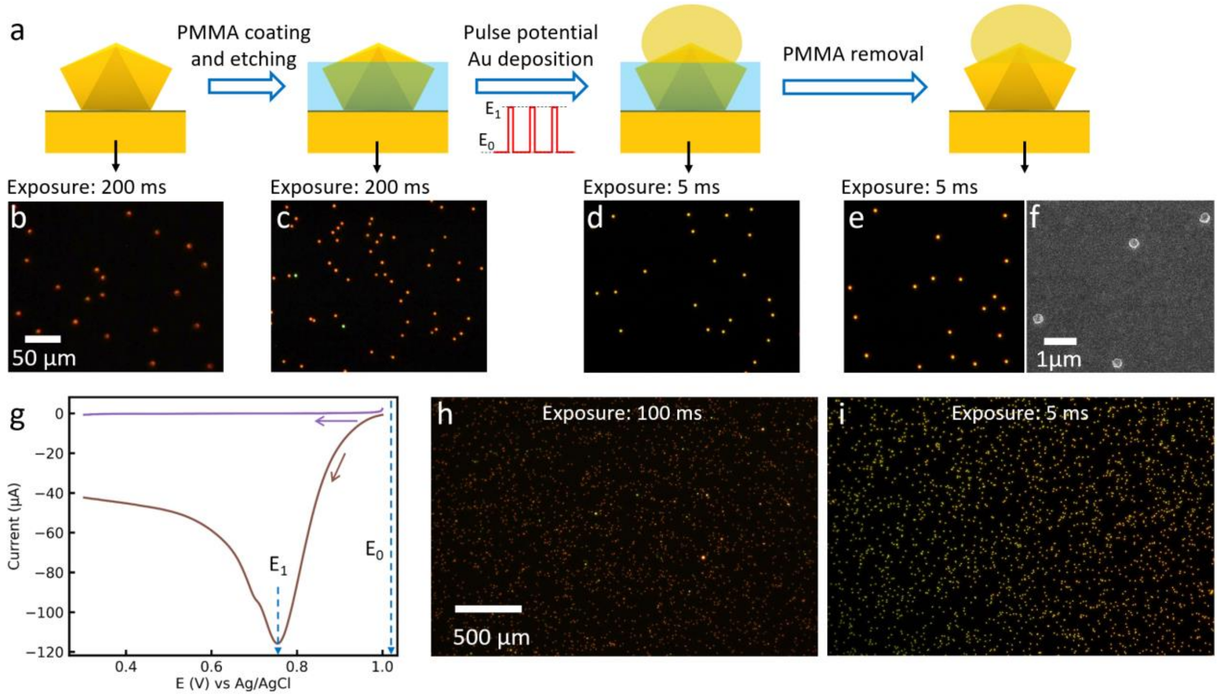

Supplementary Figure 22. Electrochemical deposition for enhancing light coupling to NDoMs. (a) Schematics of full procedures for NDoM elaboration and their corresponding (b-e) DF and (f) SEM images. The deposition time is 5 s. (g) Cyclic voltammetry (CV) curve of Au electrodes immersed in electrolytes of 0.1 M H_2SO_4 and 0.1 M H_2SO_4 + 1mM $HAuCl_4$. A large-scale view of NDoMs (h) before and (i) after Au electrodeposition.

The amount of Au deposited on NDoMs could be controlled by altering the total deposition time. Supplementary Fig. 23 shows the deposition time-dependent DF image, SERS intensity distribution, average SERS spectra and DF spectra of the nanostructures. A large scattering intensity is seen in both DF images (Supplementary Fig. 23a) and spectra (Supplementary Fig. 23e), increasing with the deposition time, indicating the deposition of Au. Deposition time dependent SERS signal enhancements (relative to NDoMs with no Au deposition) show a volcano curve (Supplementary Fig. 23d), with maximum enhancements of 26- and 5-fold for 785 nm and 633 nm excitation. The results suggest increasing height of NDoMs indeed efficiently enhances the light coupling in/out by enhancing the coupling from free space to antenna. However, the nanostructures always exceed 300 nm diameter (for the minimum deposition time of 5 s), larger than desirable for efficient coupling. A larger enhancement is expected with further optimization by reducing the size of the final geometry, but without changing their morphology and consistency.

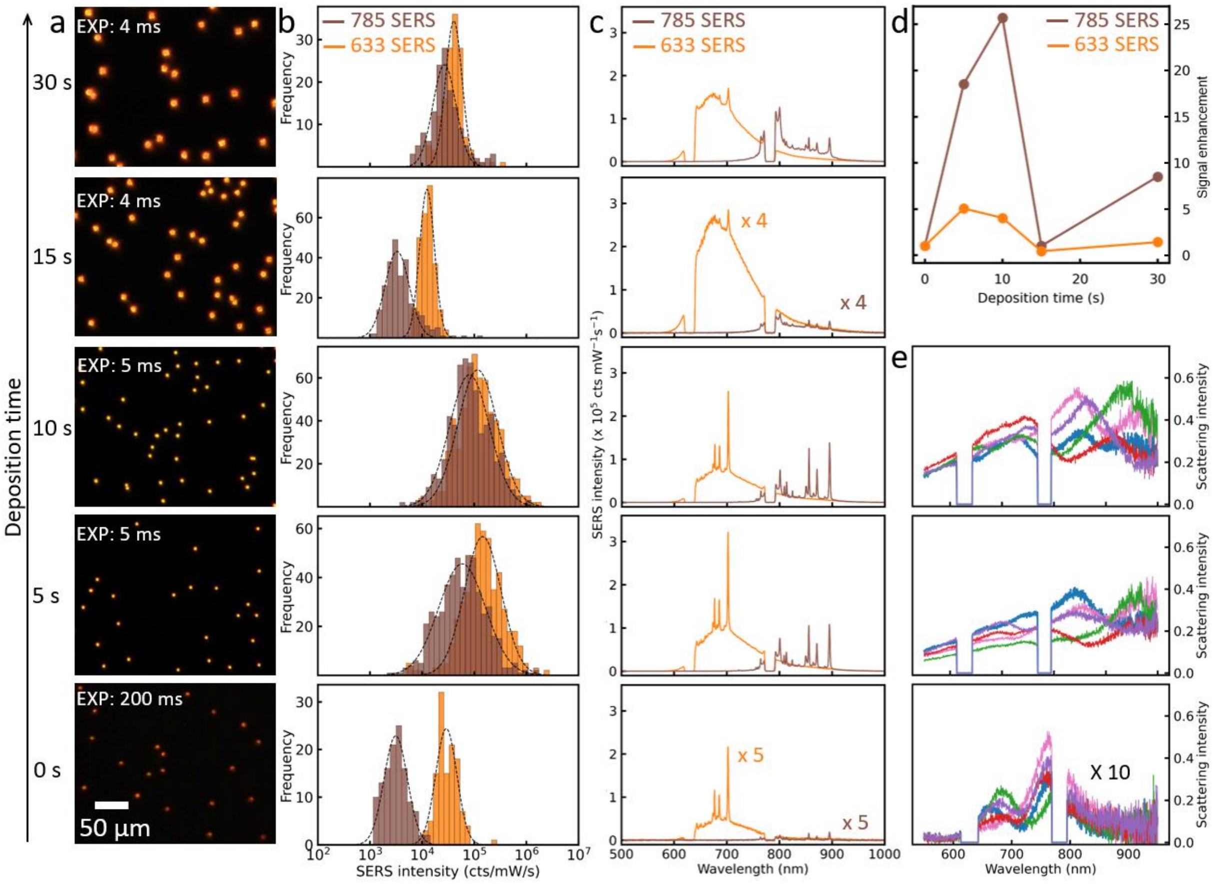

Supplementary Figure 23. Deposition time dependence. (a) DF with exposure times marked (EXP), (b) SERS intensity distribution, (c) average SERS spectra, (d) SERS enhancement, and (e) scattering spectra of several NDoMs (notch filters at laser wavelengths).

Supplementary Note 4. NDoMs elaboration using self-assembly

An alternative way to increase the height of the nanocavity assembles a single Au nanoparticle on top of each NDoM. Supplementary Fig. 24a shows the procedure of assembly using molecular crosslinkers of alkanedithiols. As in Supplementary Note 3, the NDoM substrate is first spin-coated with PMMA and etched back to expose the Au surface (15 nm above the PMMA). The substrate is then immersed in 2 mM 1,8-octanedithiol for 2 h and rinsed with a large amount of ethanol to remove the physically adsorbed dithiols. Finally, the substrate is immersed in 80 nm citrate-stabilized spherical Au nanoparticle solution for 24 h. Supplementary Fig. 24b shows the results before and after the Au nanoparticle assembly, with yield nearly zero. We observe only a few nanoparticles randomly-deposited on the substrate (Supplementary Fig. 24b, right image, small green dots). We also tried changing the concentration (from 1 to 10 mM) and length (hexanedithiol, dodecanethiol) of dithiol, the concentration and charge (citrate and CTAB) of Au nanoparticle solution, the height of the PMMA layer, and increasing ionic strength by dispersing Au nanoparticles into 0.8 mM $NaNO_3$. However, none of these strategies gives a better yield. The reason may be the PMMA on Au surface cannot be completely removed or the surface state of Au is altered by O_2 plasma, which disturbs the formation of a self-assembled monolayer of dithiols.

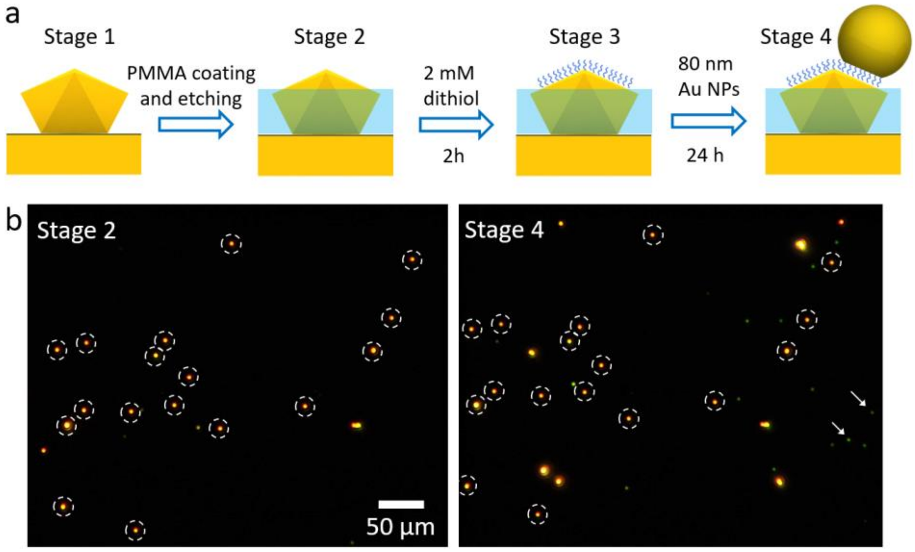

Supplementary Figure 24. Au nanoparticle assembly on NDoMs using dithiols. (a) Schematic of procedures. (b) Dark field images before (left) and after (right) nanoparticle assembly.

To avoid using O_2 plasma etching and dithiols, we used another strategy of electrostatic interactions to assemble the nanoparticles. As shown in Supplementary Fig. 25a, we first immersed the NDoMs in 15 mM CTAB solution for 2h to form a closely packed CTAB bilayer with a strong positive charge. Then, the PMMA of 30 nm is directly spin-coated on top of the substrate, which should not fully cover the NDoMs according to ref[4]. Afterwards, the substrate is incubated with citrate stabilized 80 nm Au nanoparticles (dispersed in 0.8 mM $NaNO_3$) of negative charge for 12 h to trigger the assembly. Finally, the PMMA is stripped off by rising with acetone. Although the yield is still low, we find sufficient NDoMs of the targeted structures for optical characterization. As shown in Supplementary Figs. 25b-d, 3 NDoMs (I-III) out of 37 are assembled with Au particles after 1 h, while only one (Supplementary Fig. 25d, III) is left after the PMMA is stripped off. The optical measurements show the targeted structures exhibit a huge SERS signal enhancement of > 50-fold compared with the NDoMs nearby (Supplementary Figs. 25e,f), as well as 2-fold increase in the scattering intensity. Measurements of multiple targeted nanocavities show DF spectra and SERS enhancement are of high consistency (Supplementary Figs. 25g), with SERS enhancements up to 90-fold observed.

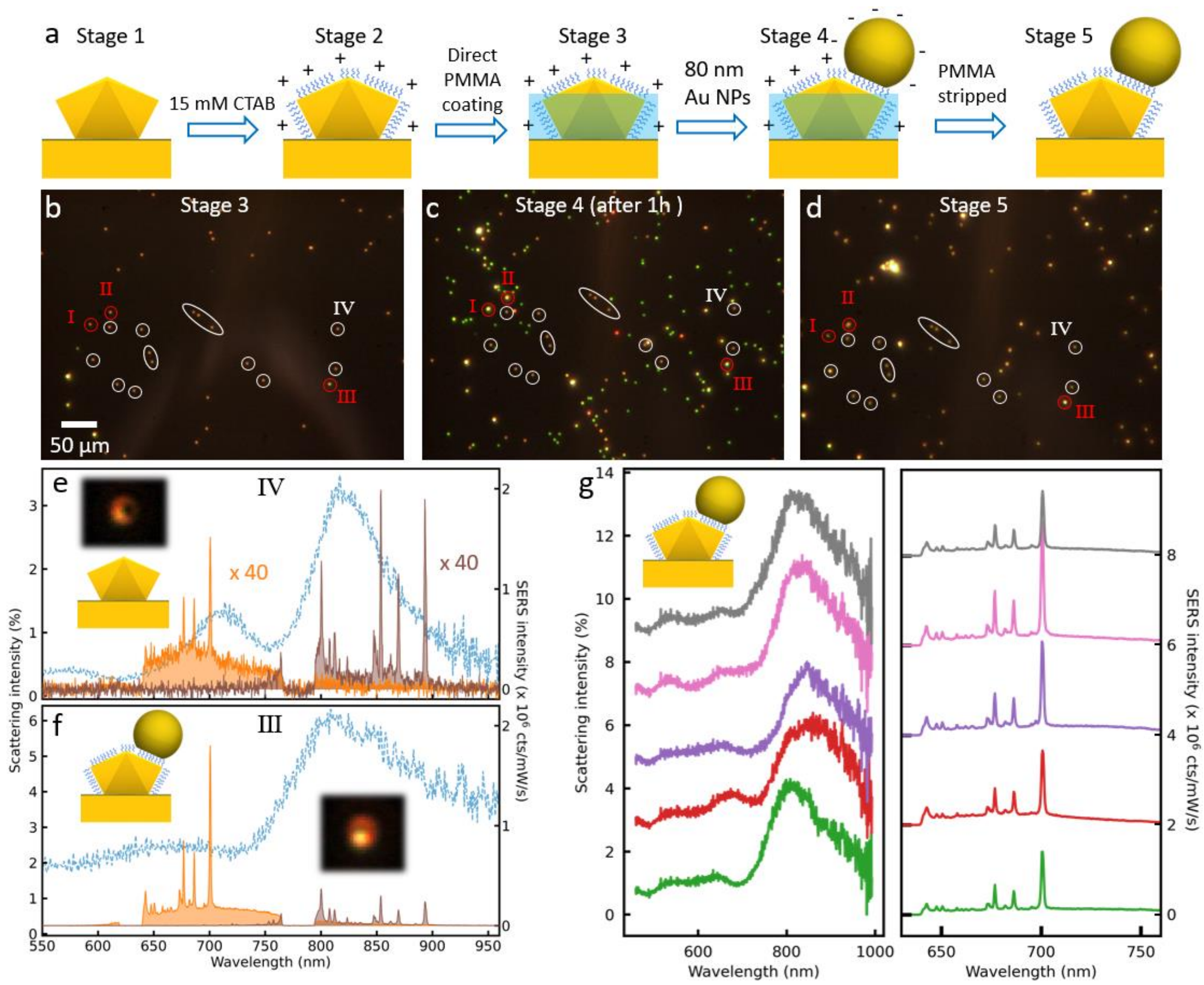

Supplementary Figure 25. Au nanoparticle assembly on NDoMs using electrostatic interaction. (a) Schematic of procedure. CTAB modification ensures a positive charge surface on NDoMs. Au nanoparticles are stabilized with citrate of negative charge. DF images of NDoMs (b, with PMMA) before and (c, with PMMA; d, PMMA stripped) after the Au nanoparticle assembly. **e,f** Comparison of DF and SERS spectra of NDoM (e) without and (f) with a Au nanoparticle on top, which are the nanostructures labelled in (b-d). The insets show the corresponding dark field images. (g) DF (left) and SERS (right) spectra of different NDoMs with Au nanoparticles assembled on top.

Reference

[1] V. Myroshnychenko, J. Nelayah, G. Adamo, N. Geuquet, J. Rodríguez-Fernández, I. Pastoriza-Santos, K. F. MacDonald, L. Henrard, L. M. Liz-Marzán, N. I. Zheludev, *Nano Lett.* **2012**, 12, 4172.

[2] M. Kamp, B. de Nijs, N. Kongsuwan, M. Saba, R. Chikkaraddy, C. A. Readman, W. M. Deacon, J. Griffiths, S. J. Barrow, O. S. Ojambati, *PNAS* **2020**, 117, 14819.

[3] X. Yao, S. Jiang, S. Luo, B.-W. Liu, T.-X. Huang, S. Hu, J. Zhu, X. Wang, B. Ren, *ACS Appl. Mater. Interfaces* **2020**, 12, 36505.

[4] S. Ye, H. Zha, Y. Xia, W. Dong, F. Yang, C. Yi, J. Tao, X. Shen, D. Yang, Z. Nie, *ACS nano* **2022**, 16, 4609.
